# GLI1 regulates a novel neuropilin-2/α6β1 integrin based autocrine pathway that contributes to breast cancer initiation

**DOI:** 10.1002/emmm.201202078

**Published:** 2013-02-21

**Authors:** Hira Lal Goel, Bryan Pursell, Cheng Chang, Leslie M Shaw, Junhao Mao, Karl Simin, Prashant Kumar, Craig W Vander Kooi, Leonard D Shultz, Dale L Greiner, Jens Henrik Norum, Rune Toftgard, Charlotte Kuperwasser, Arthur M Mercurio

**Affiliations:** 1 Department of Cancer Biology, University of Massachusetts Medical SchoolWorcester, MA, USA; 2 Department of Cellular and Molecular Biochemistry, Center for Structural Biology, University of KentuckyLexington, KY, USA; 3 The Jackson LaboratoryBar Harbor, ME, USA; 4 Program in Molecular Medicine, University of Massachusetts Medical SchoolWorcester, MA, USA; 5 Department of Bioscience and Nutrition, Center for Biosciences, Karolinska InstituteNovum, Huddinge, Sweden; 6 Department of Anatomy and Cellular Biology, Tufts University School of MedicineBoston, MA, USA

**Keywords:** breast cancer, GLI1, integrin, neuropilin-2, stem cells

## Abstract

The characterization of cells with tumour initiating potential is significant for advancing our understanding of cancer and improving therapy. Aggressive, triple-negative breast cancers (TNBCs) are enriched for tumour-initiating cells (TICs). We investigated that hypothesis that VEGF receptors expressed on TNBC cells mediate autocrine signalling that contributes to tumour initiation. We discovered the VEGF receptor neuropilin-2 (NRP2) is expressed preferentially on TICs, involved in the genesis of TNBCs and necessary for tumour initiation. The mechanism by which NRP2 signalling promotes tumour initiation involves stimulation of the α6β1 integrin, focal adhesion kinase-mediated activation of Ras/MEK signalling and consequent expression of the Hedgehog effector GLI1. GLI1 also induces BMI-1, a key stem cell factor, and it enhances NRP2 expression and the function of α6β1, establishing an autocrine loop. NRP2 can be targeted *in vivo* to retard tumour initiation. These findings reveal a novel autocrine pathway involving VEGF/NRP2, α6β1 and GLI1 that contributes to the initiation of TNBC. They also support the feasibility of NRP2-based therapy for the treatment of TNBC that targets and impedes the function of TICs.

## INTRODUCTION

The hypothesis that breast tumours harbor a population of cells that can initiate tumourigenesis is supported by strong experimental evidence (Al-Hajj et al, 2003; Baccelli & Trumpp, 2012; Gupta et al, 2009; Keller et al, 2012; Korkaya et al, 2011). The frequency of such tumour-initiating cells (TICs) is high in poorly differentiated tumours (Pece et al, 2010) and these cells may be intimately associated with an epithelial mesenchymal transition (EMT) and contribute to metastasis (DiMeo et al, 2009; Mani et al, 2008; Scheel et al, 2011). There is also evidence that luminal-like cells without basal characteristics are capable of initiating invasive breast tumours in mice (Kim et al, 2012b). Regardless of their origin, much remains to be learned about the mechanisms that enable the functions of TICs. Indeed, these cells are attractive therapeutic targets, but they are notoriously resistant to most standard chemotherapies (McDermott & Wicha, 2010). Thus, elucidating the mechanisms that contribute to the function of these cells is of obvious significance for understanding the biology of breast cancer and improving the clinical management of this disease.

A distinguishing feature of TICs is their self-sufficiency and their use of specific signalling pathways to sustain their function (DiMeo et al, 2009; Fillmore et al, 2010; Ginestier et al, 2010; Kim et al, 2012a; Korkaya et al, 2011; Marotta et al, 2011; Sansone et al, 2007; Scheel et al, 2011). Such pathways contribute to their self-renewal and de-differentiation (Scheel et al, 2011), and they are potential targets for therapeutic intervention (Korkaya et al, 2011; Marotta et al, 2011). In this context, VEGF receptors expressed on breast carcinoma cells can mediate autocrine VEGF signalling that contributes to tumour initiation and progression (Bachelder et al, 2001, 2002, 2003; Bae et al, 2008; Bagri et al, 2009; Barr et al, 2005; Bates et al, 2003; Cao et al, 2008; Castro-Rivera et al, 2004; Gray et al, 2008; Hu et al, 2007; Lichtenberger et al, 2010; Matsushita et al, 2007; Mercurio et al, 2004). These findings challenge the notion that the function of VEGF in cancer is limited to its role in angiogenesis and that therapeutic approaches based on the inhibition of VEGF and its receptors target only this function (Ferrara, 2005).

Tumour cells express tyrosine kinase VEGF receptors (VEGFR1 and VEGFR2) and neuropilins (NRPs), another family of VEGF receptors. NRP1 and NRP2 were identified initially as neuronal receptors for semaphorins, which are axon guidance factors that function primarily in the developing nervous system (Uniewicz & Fernig, 2008). The finding that NRPs can also function as VEGF receptors and that they are expressed on endothelial and tumour cells launched studies aimed at understanding their contribution to angiogenesis and tumour biology (Soker et al, 1998). NRPs have the ability to interact with and modulate the function of VEGFR1 and VEGFR2, as well as other receptors (Neufeld et al, 2002; Sulpice et al, 2008). There is also evidence that NRPs are valid targets for therapeutic inhibition of angiogenesis and cancer (Caunt et al, 2008; Goel et al, 2012a; Gray et al, 2008; Pan et al, 2007). Importantly, NRPs, functioning as VEGF receptors, have been implicated in tumour initiation and the biology of tumour stem-like cells (Beck et al, 2011; Glinka et al, 2012; Hamerlik et al, 2012). Of note, autocrine VEGF/NRP1 signalling contributes to the self-renewal of squamous skin tumours (Beck et al, 2011). Similarly, the viability, self-renewal and tumourigenicity of glioblastoma stem cells involve a VEGF/VEGFR2/NRP1 autocrine signalling loop (Hamerlik et al, 2012). However, no studies to date have implicated NRP2 in tumour initiation.

Despite the compelling evidence for the importance of VEGF/NRP signalling in tumour initiation, little is known about the mechanism by which this signalling affects the function of TICs. Moreover, the possibility that NRPs function in concert with other receptors besides the VEGFRs to drive tumour initiation has not been investigated. Here, we sought to examine the role of VEGF/NRP signalling in triple-negative breast cancers (TNBCs) because they are characterized by a high frequency of TICs (Idowu et al, 2012; Park et al, 2010) and high expression of the α6β1 integrin (Gupta et al, 2011). Clinically, TNBCs are defined by their lack of expression of the oestrogen receptor α (ERα), progesterone receptor (PR) and HER2 (ERBB2), are generally of high histological grade, poorly differentiated and more aggressive compared to other subtypes of breast cancer (Griffiths & Olin, 2012). This phenotype is consistent with the observation that NRP2 expression in human breast cancer correlates with aggressive disease and poor clinical outcome (Yasuoka et al, 2009). These features also make TNBC an ideal breast cancer sub-type to study how VEGF/NRP2 signalling functions in tandem with α6β1 to promote the initiation of breast tumours and to define the mechanism involved. Our data reveal a novel autocrine signalling pathway mediated by VEGF/NRP2 and α6β1 signalling that contributes to tumour initiation. The nexus of this pathway is the Hedgehog (Hh) effector GLI1 that is regulated by VEGF/NRP2 and that also feeds back and regulates NRP2 expression and α6β1 function.

## RESULTS

### NRP2 is associated with tumour-initiating cells and contributes to their function

**Figure 1:**
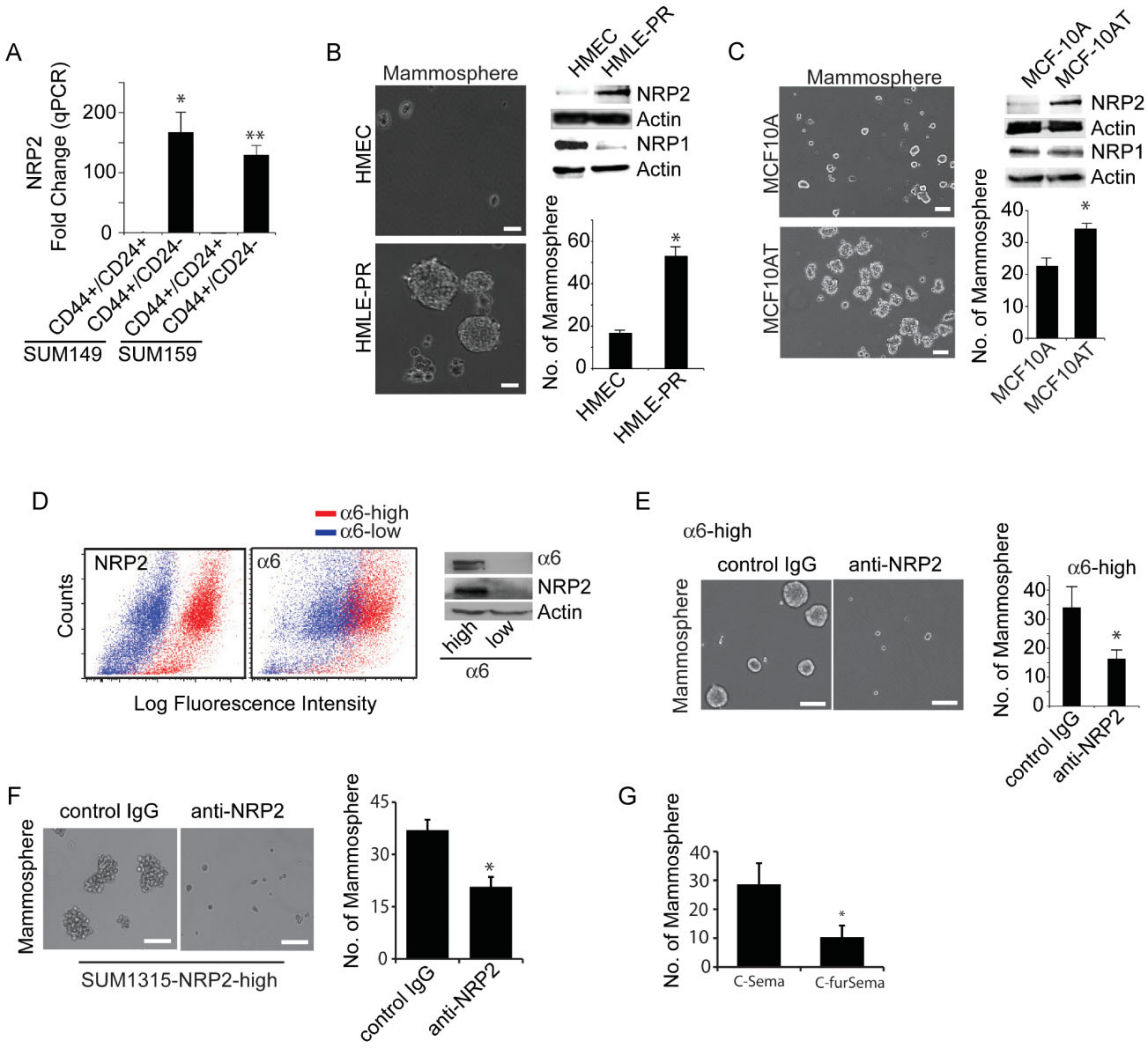
NRP2 is associated with TICs and mediates mammosphere formation. A. SUM149 and SUM159 cells were sorted by FACS into either stem (CD44^+^/CD24^−^/EpCAM^+^) or luminal (CD44^+^/CD24^+^/EpCAM^+^) populations. Expression of NRP2 was analysed using qPCR. *p = 0.0129 and **p = 0.00499. B,C. HMEC, HMLE-PR, MCF10A and MCF10AT cells were plated on low adhesion plates to measure their ability to form mammospheres. NRP2 and NRP1 expression was analysed by immunoblotting. Representative data from three independent experiments are shown. *p = 0.0032 (for B) and *p = 0.0077 (for C). D,E. Epithelial cells freshly isolated from breast cancer biopsies were sorted by FACS into either α6^high^ or α6^low^ populations. Cell extracts were immunoblotted to assess expression of NRP2, α6 and actin (D). These α6^high^ and α6^low^ populations were analysed for their ability to form mammospheres in the presence of either control IgG or a NRP2 inhibitory Ab (E). These results are consistent using three different primary tumours (*p = 0.0036). F. The NRP2^high^ population was sorted from SUM1315 cells by FACS and analysed for their ability to form mammospheres in the presence of either control IgG or a NRP2 inhibitory Ab (*p = 0.0049, three independent experiments). G. The NRP2^high^ and NRP2^low^ populations of SUM1315 were isolated. The ability of the NRP2^high^ population to form mammospheres in the presence of a VEGF-NRP inhibitory peptide (C-furSema) or control peptide (C-Sema) was determined. Representative data from three independent experiments are shown (*p = 0.028). Scale bar = 100 µm for all panels. For all panels, error bars represent the mean ± SD. Statistical differences between data groups were determined using Student's *t*-test.

Given that TICs involved in TNBC are characterized by high expression of the α6β1 integrin (CD49f) (Meyer et al, 2010) and our previous finding that this integrin associates with NRP2 in TNBC (Goel et al, 2012b), we examined the expression and function of NRP2 in this population of cells. Initially, we compared NRP2 expression in sorted populations of TNBC cell lines: SUM149 and SUM159. The CD44^+^/CD24^−^/EpCAM^+^ population sorted from these lines has stem-like properties and is able to initiate tumour formation much more readily than the luminal population (CD44^+^/CD24^+^/EpCam^+^) (Fillmore & Kuperwasser, 2008). Indeed, the stem-like CD44^+^/CD24^−^/EpCAM^+^ population expresses very high levels of NRP2 compared to the luminal population (Fig 1A). This stem-like population also exhibits high expression of the α6β1 integrin (Gupta et al, 2011). Subsequently, we assessed NRP2 expression in normal mammary epithelial cells that had been transformed with specific oncogenes: HMEC transformed with large T antigen, hTERT and Ras (HMLE-PR) and MCF10A cells transformed with T24 H-ras (MCF10A-AT). Oncogenic transformation induced NRP2 expression in both cell types concomitant with an increase in mammosphere formation (Fig 1B and C), but it did not increase NRP1 expression (Fig 1B and C).

To investigate the role of NRP2 in TICs and its relationship to α6β1 further, we used primary tumour cells isolated from freshly resected breast tumour biopsies. These epithelial cells were sorted into α6^high^ and α6^low^ populations (Fig 1D and Supporting Information Fig S1A). The α6^high^ population, which expressed high NRP2 (Fig 1D), formed mammospheres significantly more than the α6^low^ population (Supporting Information Fig S1B). Importantly, mammosphere formation was inhibited by a NRP2 blocking antibody (Fig 1E).

**Figure 2:**
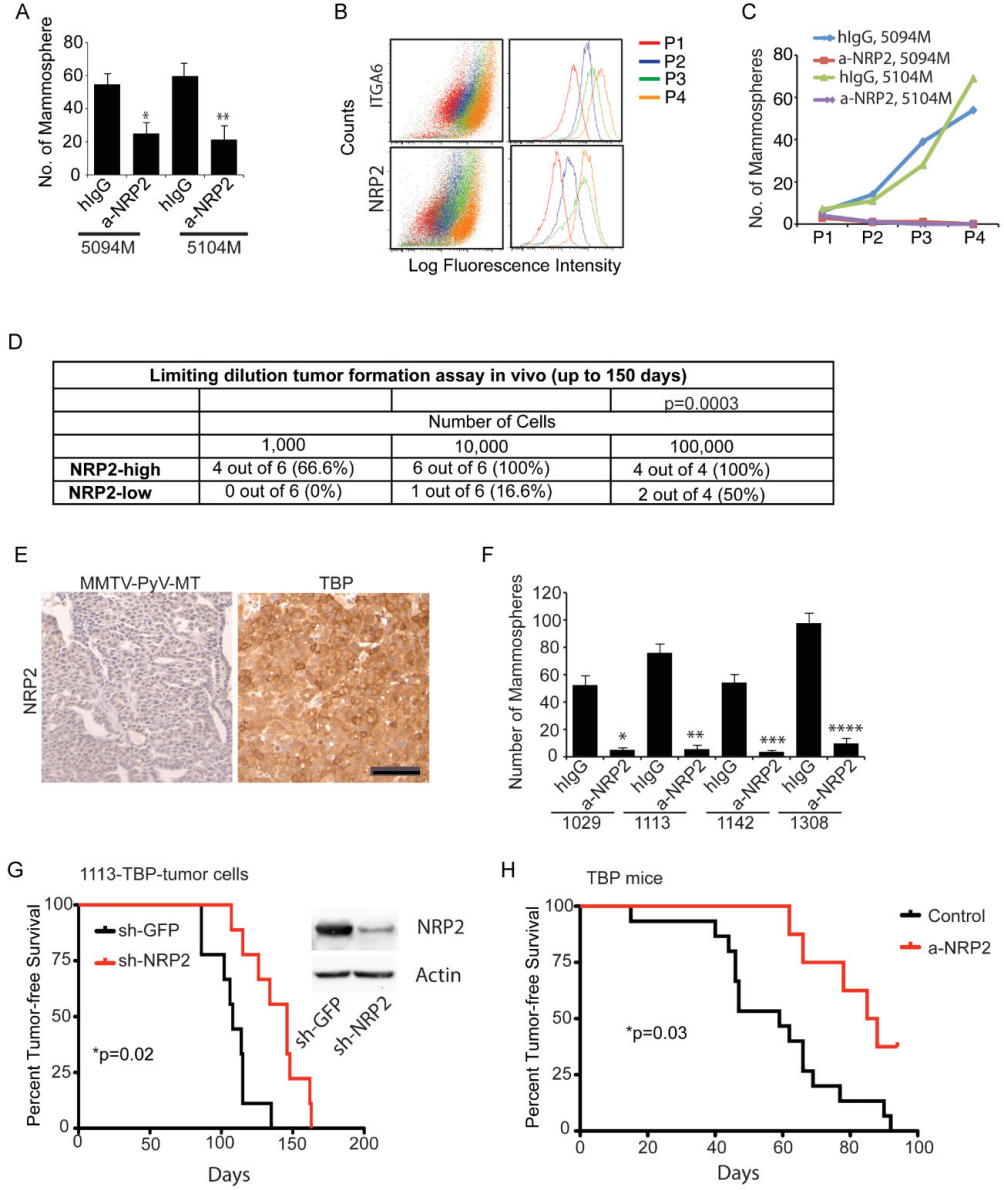
NRP2 mediates self-renewal and contributes to tumour initiation A. Freshly harvested breast tumour cells from ascites (5104M and 5094M) were assayed for mammosphere formation in the presence of either control IgG or a NRP2 Ab. Representative data from three independent experiments are shown (*p = 0.0051; **p = 0.00431). B. Mammospheres from 5094M and 5104M cells were passaged serially up to four times and the surface expression of ITGA6 and NRP2 was quantified by flow cytometry for each passage. C. Mammospheres from 5094M and 5104M cells were passaged serially in the presence of either hIgG or a NRP2. D. The NRP2^high^ and NRP2^low^ populations of SUM1315 cells were transplanted into the mammary fat pads of NSG mice using 10-fold serial dilution. The formation of palpable tumours was used to evaluate tumour initiation. The summary chi-square statistic with one degree of freedom is 12.81 and the p-value is 0.0003, indicating that NRP2^high^ cells form significantly more tumours than NRP2^low^ cells. E. Formalin-fixed and paraffin-embedded sections from either TBP or MMTV-PyV-MT mouse mammary tumours (*n* = 3) were stained using a NRP2 Ab. Scale bar = 25 µm. F. Primary cells isolated from four independent TBP tumours were used to assay mammosphere formation in the presence of either control IgG or a NRP2 Ab. Representative data from three independent experiments are shown. *p = 0.01; **p = 0.003; ***p = 0.011; ****p = 0.0015. G. Primary cells isolated from TBP mammary tumours that had been infected with either NRP2 or GFP shRNAs were transplanted into the mammary fat pads of FVB mice (1 × 10^5^ cells per injection; 9 mice per group). The formation of palpable tumours was used to evaluate tumour initiation. Immunoblots show downregulation of NRP2. The curve comparison was done using the Log-rank test. *p = 0.02. H. Pregnant female TBP mice either control (*n* = 15) or were injected i.p. with Anti-Nrp2^B^ (twice weekly; *n* = 8). A total of four injections were given. The formation of palpable tumours was used to evaluate tumour initiation. The curve comparison was done using Log-rank test. *p = 0.03. Error bars represent the mean ± SD for the *in vitro* experiments. Statistical differences between data groups were determined using Student's *t*-test.

Additional evidence to implicate NRP2 in mammosphere formation was obtained using SUM1315 cells, which have a triple-negative phenotype. These cells were sorted into NRP2^high^ and NRP2^low^ populations (Fig 1F and Supporting Information Fig S1C and D). Approximately 10% of SUM1315 cells exhibit high NRP2 expression, which is similar to the frequency observed in freshly isolated breast tumour cells [Supporting Information Fig S1C (Goel et al, 2012b)]. The NRP2^high^ population formed mammospheres significantly more than did the NRP2^low^ population (Supporting Information Fig S1E) and a NRP2 Ab blocked mammosphere formation (Fig 1F). These results were substantiated by the use of an inhibitory peptide, C-furSEMA (Parker et al, 2010). This peptide, which blocks the interaction of VEGF with NRPs specifically, inhibited mammosphere formation by SUM1315 NRP2^high^ cells (Fig 1G). We also established two cell lines from the ascites fluid of breast cancer patients. Both of these cell lines are able to form tumours *in vivo*. NRP2 is necessary for these cells to form mammospheres based on the use of a function blocking Ab (Fig 2A). Importantly, mammospheres formed from these cells can be passaged serially up to five times and there is an increase in the expression of NRP2 and α6β1 integrin with each passage (Fig 2B) suggesting a potential role for NRP2 in self-renewal. Moreover, cells cultured in the presence of the NRP2 Ab failed to form any mammospheres after the second passage (Fig 2C).

The involvement of NRP2 in tumour initiation was evaluated *in vivo* using the NRP2^high^ and NRP2^low^ populations sorted from SUM1315 cells. The ability of these populations to initiate tumours in the mammary fat pad was compared by limiting dilution. The NRP2^high^ population formed tumours much more readily than did the NRP2^low^ population (Fig 2D). A significant fraction of mice (66.6%) injected with only 10^3^ NRP2^high^ cells formed tumours in contrast to the lack of tumour formation in mice injected with the same number of NRP2^low^ cells (Fig 2D). We made use of a recently described mouse model of triple-negative breast carcinoma in which the Rb, p53 and BRCA1 pathways were inactivated in the mammary epithelium using a transgene encoding a fragment of the SV40 Large T-antigen (T121) to inactivate pRb, along with conditional alleles of p53 and Brca1. These TgMFT121; Brca1f/f p53f/f; TgWAP-Cre mice (hereafter referred to as TBP (T121, BRCA1, p53)), and they develop highly penetrant, metastatic adenocarcinomas with a triple-negative phenotype (Kumar et al, 2012). These tumours, in marked contrast to mouse mammary tumour virus (MMTV)-PyV-MT tumours, express abundant NRP2 (Fig 2E). Cells derived from TBP tumours form mammospheres that are dependent upon NRP2 (Fig 2F). Depletion of NRP2 in TBP tumour cells using shRNA significantly attenuated their ability to form tumours in mice (Fig 2G). To establish the role of NRP2 in tumour initiation more definitively, we injected TBP mice with a NRP2 inhibitory Ab (Anti-Nrp2^B^) at the time of pregnancy, which triggers tumour onset in this transgenic model. As shown in Fig 2H, the onset of these tumours was significantly delayed by NRP2 inhibition.

### VEGF/NRP2 and α6β1 contribute to FAK-mediated regulation of BMI-1

**Figure 3:**
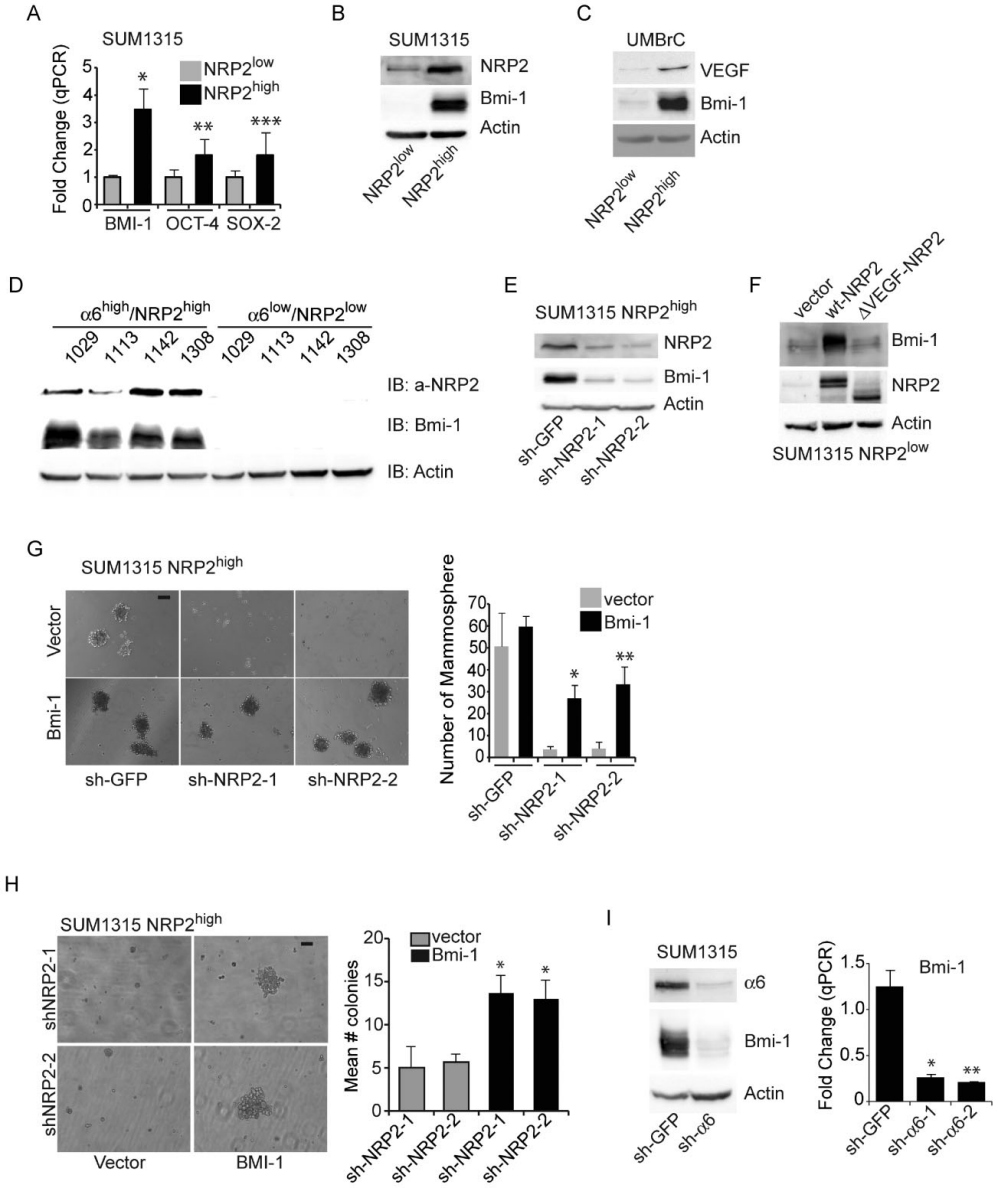
VEGF/NRP2 signalling promotes mammosphere formation by regulating BMI-1 A,B. Expression of BMI-1, OCT-4 and SOX-2 was quantified by qPCR in the NRP2^high^ and NRP2^low^ populations of SUM1315 cells and the expression pattern of BMI-1 was verified by immunoblotting. Representative data from three independent experiments are shown. *p = 0.15; **p = 0.05; ***p = 0.04. C. Expression of VEGF, BMI-1 and actin was compared by immunoblotting in the NRP2^high^ and NRP2^low^ populations isolated from breast cancer biopsies. These results are consistent using three different biopsies. D. Cells isolated from four independent TBP tumours were sorted by FACS into either α6^high^/NRP2^high^ or α6^low^/NRP2^low^ populations. Cell extracts from these subpopulations were immunoblotted to measure expression of NRP2, BMI-1 and actin. E. NRP2 expression was depleted by shRNAs in NRP2^high^ SUM1315 subpopulation and BMI-1 expression was assessed by immunoblotting. Representative data from three independent experiments are shown. F. Either wild-type NRP2 or ΔVEGF-NRP2 constructs were expressed in the NRP2^low^ SUM1315 subpopulation and the effect on BMI-1 expression was determined by immunoblotting. Representative data from two independent experiments are shown. G. Either a control vector or a BMI-1 containing vector was expressed in the NRP2^high^ SUM1315 subpopulation that had been depleted of NRP2 expression and the ability of these cells to form mammospheres was assayed. *p = 0.023; **p = 0.026 (two independent experiments). H. The same populations of cells described in (G) were used to measure growth in soft agar. *p = 0.019; **p = 0.029 (two independent experiments). I. SUM1315 cells were transfected with either α6 integrin or GFP shRNAs and the expression of α6, BMI-1 and actin was analysed by immunoblotting (left panel). BMI-1 mRNA expression was quantified in these by qPCR (right panel). Representative data from three independent experiments are shown (*p = 0.007; **p = 0.0025). Scale bar = 100 µm for all panels. Error bars represent the mean ± SD for all panels. Statistical differences between data groups were determined using Student's *t*-test.

The involvement of NRP2 in tumour initiation suggests that its function may be linked to specific stem cell factors. Comparison of the NRP2^high^ and NRP2^low^ populations sorted from SUM1315 cells revealed elevated expression of BMI-1, OCT-4 and SOX-2 in the NRP2^high^ population, although BMI-1 exhibited the largest difference in expression between NRP2^high^ and NRP2^low^ populations (Fig 3A and B). The relationship between NRP2 and BMI-1 was further confirmed using NRP2^high^ and NRP2^low^ sorted populations from freshly isolated breast tumours (Fig 3C). Moreover, the α6^high^/NRP2^high^ population sorted from the TBP transgenic cells exhibited markedly higher BMI-1 expression than did the α6^low^/NRP2^low^ population (Fig 3D).

NRP2 is necessary for BMI-1 expression (Fig 3E), consistent with our previous finding in prostate cancer (Goel et al, 2012a). To investigate the role of VEGF in regulating BMI-1 expression, either wild-type NRP2 or a NRP2 mutant lacking the b1 and b2 domains, which mediate VEGF binding (Geretti et al, 2008), was expressed in the SUM1315 NRP2^low^ population. This mutant construct was unable to induce BMI-1 expression in contrast to the wild-type construct (Fig 3F), providing evidence for the requirement of autocrine VEGF signalling in regulating BMI-1.

The contribution of BMI-1 to mammosphere formation was assessed by depleting NRP2 expression in the SUM1315 NRP2^high^ population, which inhibited mammospheres, and subsequently rescuing their formation by exogenous BMI-1 expression (Fig 3G). Similar results were obtained in soft agar assays (Fig 3H), suggesting a role for the NRP2/BMI-1 pathway in survival of cells in an anchorage-independent manner.

Recently, we reported that VEGF/NRP2 signalling regulates the function of the α6β1 integrin (Goel et al, 2012b) and VEGF/NRP2 signalling enables the α6β1 integrin and its ability to activate focal adhesion kinase (FAK). We also reported that the ability of VEGF/NPR2 to induce BMI-1 expression is FAK-dependent in prostate cancer (Goel et al, 2012a). These findings are of particular interest since high expression of α6β1 (CD49f) characterizes many tumour stem-like cells including those of the breast (Friedrichs et al, 1995; Honeth et al, 2008; Lathia et al, 2010; Mulholland et al, 2009; Schober & Fuchs, 2011; Vieira et al, 2012). Indeed, loss of α6β1 depleted BMI-1 mRNA and protein expression in SUM1315 cells (Fig 3I).

**Figure 4:**
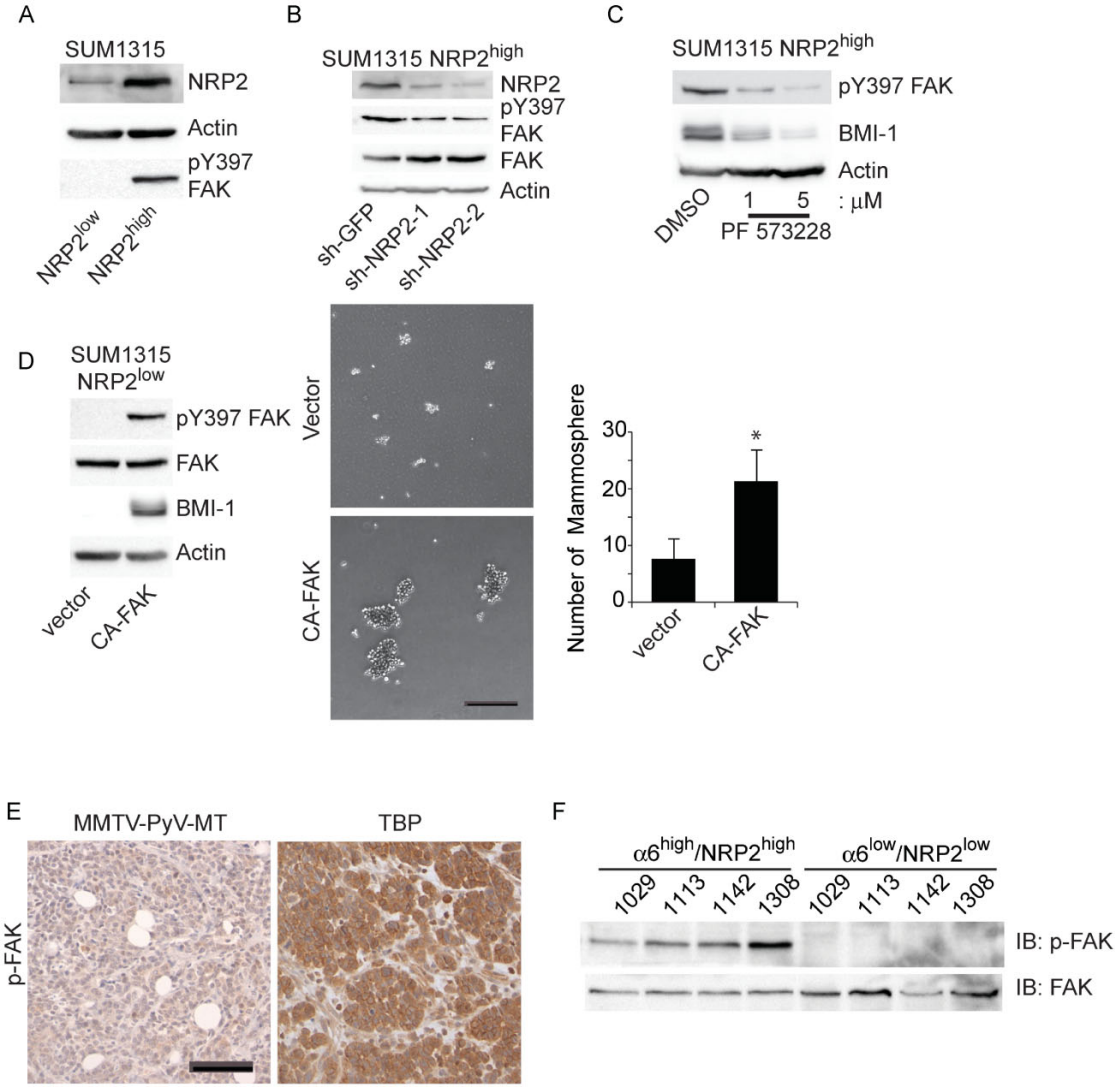
FAK mediates the ability of NRP2 to promote mammosphere formation A. Extracts from the NRP2^high^ and NRP2^low^ populations of SUM1315 cells were immunoblotted with Abs specific for NRP2, p-FAK (pY397) or actin. B. NRP2 expression was depleted by shRNAs in NRP2^high^ SUM1315 population and FAK phosphorylation (pY397) expression was assessed by immunoblotting. C. The NRP2^high^ SUM1315 population was treated with a FAK inhibitor (PF-573228) and the effect on BMI-1 expression was determined by immunoblotting. D. A constitutively active FAK construct was expressed in the NRP2^low^ population of SUM1315 cells and the effect on BMI-1 expression (left panel) and mammosphere formation (middle and right panels) was determined. The above experiments were repeated at least twice. Scale bar = 100 µm. Error bar represents the mean ± SD. Statistical differences between data groups were determined using Student's *t*-test. *p = 0.029. E. Formalin-fixed and paraffin-embedded sections from either TBP or MMTV-PyV-MT mouse mammary tumours (*n* = 4) were stained using an Ab specific for phosphorylated FAK (pY397). Scale bar = 25 µm. F. Cells isolated from four independent TBP tumours were sorted by FACS into either a α6^high^/NRP2^high^ or α6^low^/NRP2^low^ populations and immunoblotted to determine the relative expression of p-FAK and FAK.

In addition, we observed that the SUM1315 NRP2^high^ population exhibited significantly higher levels of activated FAK (pY397) than did the SUM1315 NRP2^low^ population (Fig 4A) and that this FAK activation is dependent on NRP2 (Fig 4B). Inhibition of FAK kinase activity diminished BMI-1 expression (Fig 4C) and expression of constitutively active FAK in the SUM1315 NRP2^low^ population induced FAK activation and BMI-1 expression (Fig 4D). Importantly, expression of constitutively active FAK increased the ability of these cells to form mammospheres significantly (Fig 4D). Furthermore, expression of pY397 FAK is markedly elevated in TBP transgenic tumours compared to MMTV-PyV-MT tumours, and the α6^high^/NRP2^high^ population sorted from TBP tumour cells exhibited markedly higher FAK activation than did the α6^low^/NRP2^low^ population (Fig 4E–F).

### GLI1 is an effector of VEGF/NRP2 and α6β1 signalling that regulates BMI-1

**Figure 5:**
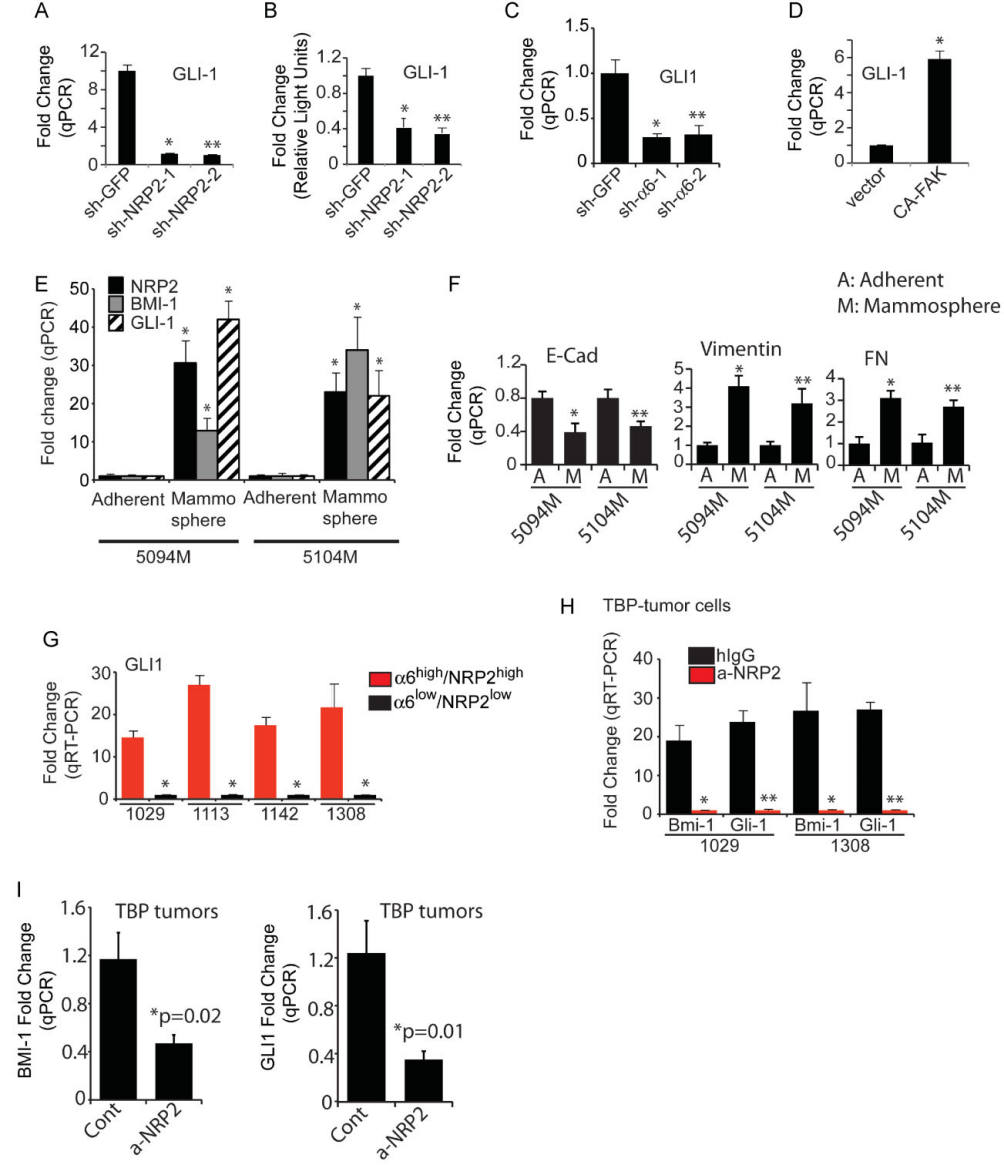
NRP2 induces expression of GLI1 A,B. NRP2 expression was depleted in SUM1315 and the effect on GLI1 mRNA expression was quantified by qPCR (A: *p = 0.015; **p = 0.01) and on GLI1 promoter activity by a luciferase reporter assay (B: *p = 0.0045; **p = 0.005). C. Expression of the α6 integrin in SUM1315 cells was diminished by shRNA and the effect on GLI1 mRNA expression was quantified by qPCR. *p = 0.01; **p = 0.004 (three independent experiments). D. A constitutively active FAK construct was expressed in the NRP2^low^ population of SUM1315 cells the effect on GLI1 mRNA expression was quantified by qPCR. The above experiments were repeated at least twice (*p = 0.002). E,F. mRNA expression of NRP2, GLI1, BMI-1 (E) and E-cadherin, vimentin and fibronectin (F) was compared between adherent and mammosphere cultures of 5094M and 5104M cells, which were isolated from ascites fluid. For panel E, the p-values for 5094M are 0.01 (NRP2), 0.02 (BMI-1), 0.01 (GLI1); and for 5104M they are 0.015 (NRP2), 0.02 (BMI-1), 0.02 (GLI1). For panel F, *p*-values are *0.01, **0.01 (for E-Cad); *0.006, **0.03 (for vimentin); *0.001, **0.004 (for fibronectin). G. Cells isolated from four independent TBP tumours were sorted by FACS into either α6^high^/NRP2^high^ or α6^low^/NRP2^low^ populations and GLI1 expression in these populations was quantified by qPCR. *p = 0.004 (for 1029); *p = 0.002 (for 1113); *p = 0.003 (for 1142); *p = 0.02 (for 1308). H. Tumour cells isolated from two independent TBP tumours were plated on low-attachment plate to form mammospheres in the presence of either Anti-Nrp2^B^ or control IgG. GLI1 and BMI-1 expression in these mammospheres was quantified by qPCR. *p = 0.01,**p = 0.005 (for 1029); *p = 0.025, **p = 0.001 (for 1308). I. Tumour extracts from control or NRP2-antibody-treated TBP females were analysed for expression of GLI1 and BMI-1 by qPCR. Tumours from three independent mice from each group were analysed. Error bars represent the mean ± SD for all panels. Statistical differences between data groups were determined using Student's *t*-test.

To determine the mechanism by which the activation of FAK by VEGF/NRP2 and α6β1 induces BMI-1, we focused on GLI1, a Hh pathway effector molecule because it is highly expressed in normal human mammary stem/progenitor cells and contributes to mammosphere formation, and the finding that GLI1 regulates BMI-1 expression (Leung et al, 2004; Liu et al, 2006). Moreover, the hedgehog/GLI1 pathway contributes to the function of tumour-initiating cells in other cancer types (Clement et al, 2007; Peacock et al, 2007; Stecca et al, 2007; Varnat et al, 2009). We discovered that depletion of NRP2 in SUM1315 cells diminished GLI1 expression (Fig 5A), as well as its promoter activity as assessed by a luciferase reporter construct (Fig 5B). Unexpectedly, we found that GLI1 expression was also dependent upon expression of α6β1 integrin (Fig 5C) and FAK activation (Fig 5D). This signalling pathway was confirmed in freshly isolated ascites cell lines (5094M and 5104M cells), because expression of NRP2, BMI-1 and GLI1 was much higher when these cells were cultured as mammospheres as compared to adherent cells (Fig 5E). Interestingly, mammosphere culture promotes an EMT phenotype as evidenced by the decreased expression of E-cadherin and increased expression of vimentin and fibronectin compared to adherent cells (Fig 5F). These findings were substantiated by demonstrating that GLI1 expression is dramatically higher in the α6^high^/NRP2^high^ population sorted from TBP tumour cells compared to the α6^low^/NRP2^low^ population (Fig 5G). Similarly, antibody-mediated inhibition of NRP2 reduced the expression of BMI-1 and GLI1 in TBP tumour cell lines (Fig 5H), as well as in primary tumours isolated from TBP mice described in Fig 2H (Fig 5I).

**Figure 6:**
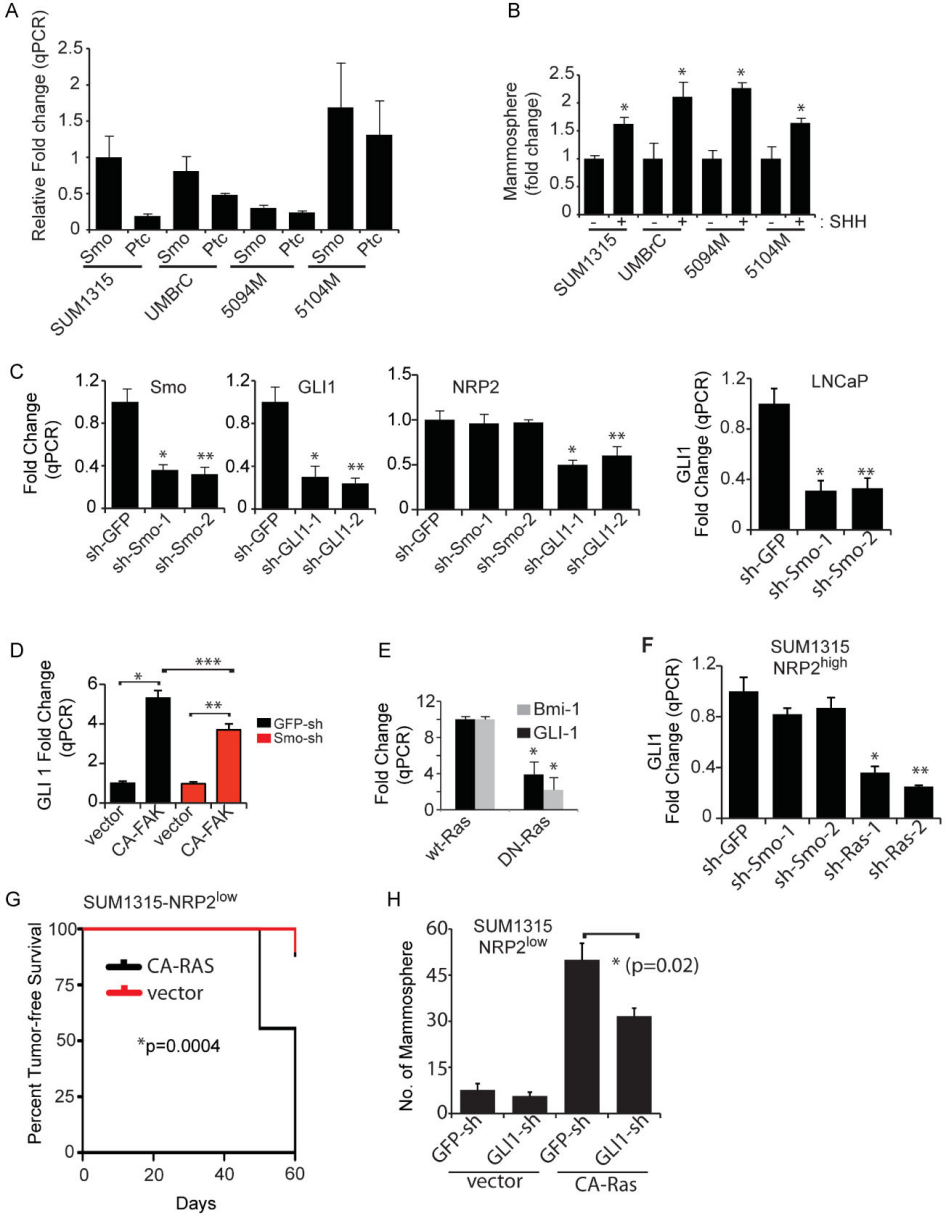
NRP2 induction of GLI1 is mediated by non-canonical FAK/Ras signalling A. Smoothened and Patched mRNA expression was quantified in SUM1315, UMBrC, 5094M and 5104M cells. Fold change was calculated relative to the value for Smoothened expression in SUM1315 cells, which was set at 1. B. These cells were stimulated with Shh conditioned media and the effect on their ability to form mammospheres was quantified. *p = 0.005 (for SUM1315); *p = 0.01 (for UMBrC); *p = 0.001 (for 5094M); *p = 0.036 (for 5104M). C. The expression of Smoothened and GLI1 was diminished in SUM1315 cells using shRNAs and the impact on NRP2 mRNA expression was quantified by qPCR. *p = 0.008, **p = 0.005 (for Smo); *p = 0.003, **p = 0.02 (for GLI1); *p = 0.004, **p = 0.008 (for NRP2). The effect of Smoothened down-regulation on GLI1 mRNA expression was also measured using qPCR in LNCaP cells as a positive control (*p = 0.001; **p = 0.001). D. The expression of Smoothened was diminished in SUM1315 cells using shRNAs and the effect of CA-FAK expression on GLI1 mRNA expression was quantified by qPCR (*p = 0.0039; **p = 0.0047; ***p = 0.003). E. Wild-type and dominant negative Ras (DN-Ras) constructs were expressed in SUM1315 cells and the effect on BMI-1 and GLI1 mRNA expression was quantified by qPCR. *p = 0.01 (for GLI1); *p = 0.007 (for BMI-1). F. The effect of Smoothened or Ras down-regulation on GLI1 mRNA expression was measured using qPCR. *p = 0.0006; **p = 0.007. G. The NRP2^low^ population of SUM1315 cells that expressed either a vector control or a CA-Ras construct (8 mice per group) was implanted in the mammary fat pads of NSG mice and tumour formation was assessed by palpation. The curve comparison was done using Log-rank test (*p = 0.0004). H. The effect of GLI1 down-regulation in cells expressing either vector or a CA-Ras construct on mammosphere formation was measured (*p = 0.02). Error bars represent the mean ± SD for the *in vitro* experiments. Statistical differences between data groups were determined using Student's *t*-test.

The issue of whether GLI1 is induced by canonical or non-canonical Hh signalling in our models was addressed initially by examining the expression of Patched and Smoothened in both SUM1315 and the cells that we isolated from human breast tumours. All of these cells express Patched and Smoothened (Fig 6A) and exogenous Shh increased their ability to form mammospheres (Fig 6B). However, down-regulation of Smoothened did not impact NRP2 expression, in contrast to down-regulation of GLI1, suggesting that a non-canonical pathway is the major driver of this autocrine loop (Fig 6C). Smoothened down-regulation did reduce GLI1 expression in LNCaP prostate cancer cells proving the efficacy of the smoothened shRNAs (Fig 6C). The finding that activated FAK increased GLI1 expression also supports non-canonical Hh signalling (Fig 6D).

To define the signalling pathway connecting NRP2/VEGF and integrin/FAK signalling to expression of GLI1 and BMI-1, we investigated the role of Ras because Ras is known to regulate non-canonical GLI1 activation (Lauth & Toftgard, 2007; Stecca et al, 2007; Varnat et al, 2009). Moreover, Ras is a downstream effector of integrin/FAK signalling (Guan, 2010; Schaller, 2010) supporting our finding that integrin (α6β1) signalling regulates GLI1 expression (Fig 5C). Indeed, expression of a dominant negative form of Ras in the SUM1315 NRP2^high^ population reduced expression of GLI1 and BMI-1 significantly (Fig 6E), and knock-down of Ras itself but not Smoothened reduced GLI1 expression (Fig 6F). The role of NRP2-mediated Ras activation in tumour initiation is supported by the finding that expression of a constitutively-active Ras construct in NRP2^low^ SUM1315 cells promoted tumour formation (Fig 6G). To assess the contribution of GLI1 in Ras-induced mammosphere formation, we down-regulated GLI1 expression in NRP2^low^ SUM1315 cells expressing constitutively-active Ras and observed a significant inhibition of mammosphere formation (Fig 6H).

**Figure 7:**
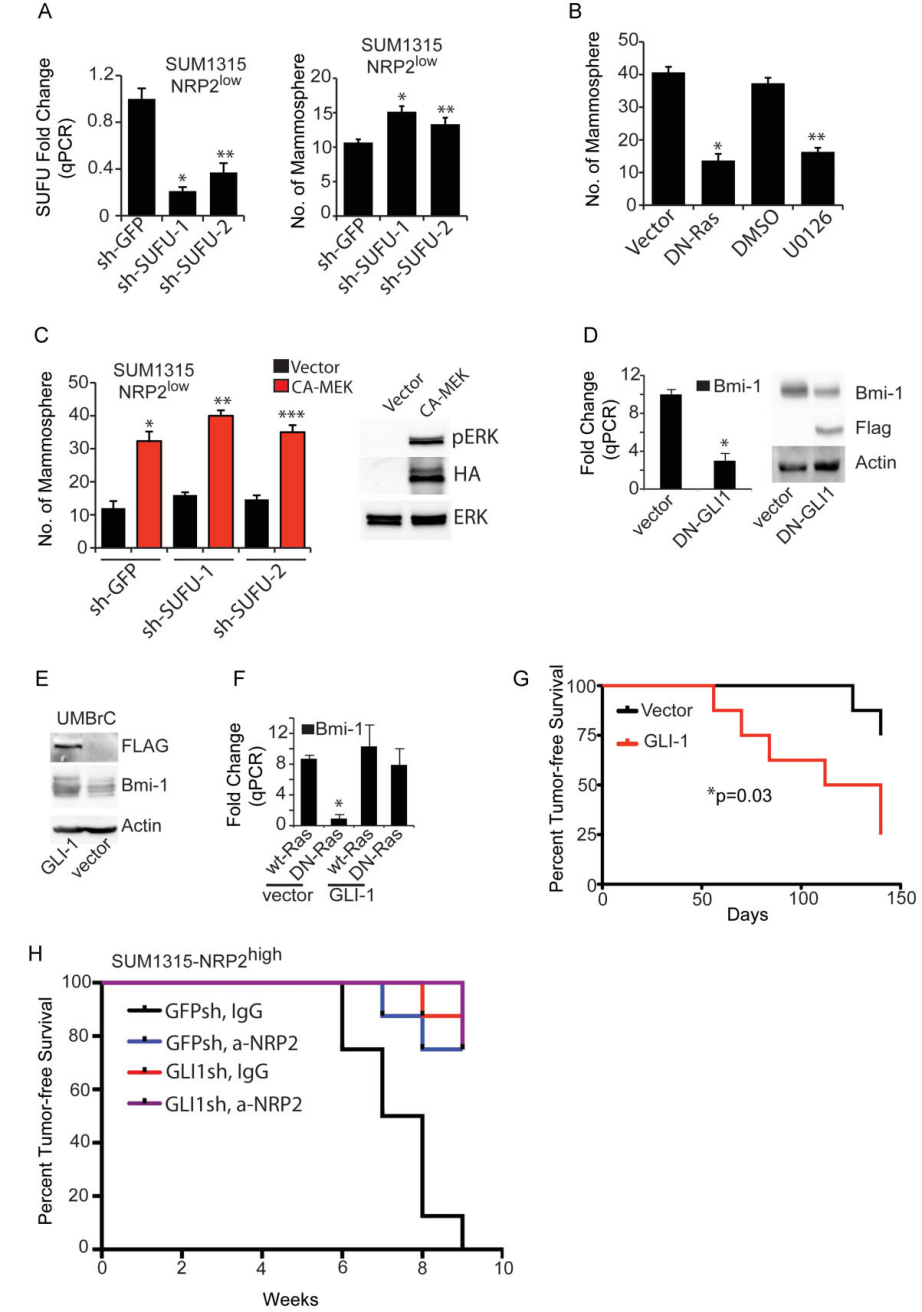
NRP2/Ras regulate GLI1 predominantly by the ERK pathway. A. The effect of SUFU downregulation on SUFU mRNA expression (*p = 0.001; **p = 0.001) and mammosphere formation (*p = 0.01; **p = 0.03) was measured in the NRP2^low^ population of SUM1315 cells. B. The NRP2^high^ population of SUM1315 cells was either transfected with DN-Ras or treated with the MEK inhibitor U0126 and mammosphere assays were performed (*p = 0.0001; **p = 0.0002). C. The NRP2^low^ population of SUM1315 cells expressing either GFPsh or SUFU-sh was transfected with either control vector or CA-MEK and mammosphere assays were performed. Immunoblot shows expression of HA-tagged CA-MEK (*p = 0.001; **p = 0.0003; ***p = 0.001). D. A Flag-tagged dominant negative GLI1 construct (DN-GLI1) was expressed in SUM1315 cells and the effect on BMI-1 mRNA and protein expression was determined (*p = 0.001). E. A GLI1 construct was expressed in the NRP2^low^ population of epithelial cells freshly isolated from breast cancer biopsies (UMBrC) and the effect on BMI-1 expression was measured by immunoblotting (*p = 0.0003). F. A GLI1 construct was expressed in the NRP2^low^ population of SUM1315 cells that also expressed either wild-type or DN-Ras, and the effect on BMI-1 mRNA expression was quantified by qPCR. G. The NRP2^low^ population of SUM1315 cells that expressed either a vector control or a GLI1 construct (8 mice per group) were implanted in the mammary fat pads of NSG mice and tumour formation was assessed by palpation. The curve comparison was done using the Log-rank test (*p = 0.03). H. The NRP2^high^ population of SUM1315 cells that expressed either GFP-sh or GLI1-sh was implanted in the mammary fat pads of NSG mice and treated with IgG or a-NRP2 (8 mice per group). Tumour formation was assessed by palpation. The curve comparison was done using the Log-rank test (*p = 0.0001). Error bars represent the mean ± SD for the *in vitro* experiments. Statistical differences between data groups were determined using Student's *t*-test.

The possibility existed that FAK induces GLI1 expression by inhibiting Suppressor of Fused (SuFu), a negative regulator of GLI1. This possibility is supported by the observations that the regulation of GLI1 by NRPs is dependent on SuFu (Hillman et al, 2011) and that oncogenic Ras enhances GLI1 activation by suppressing SUFU (Kasai et al, 2008). However, down-regulation of SUFU increased mammosphere formation only slightly (Fig 7A) indicating the involvement of other Ras signalling pathways. One of the major signalling pathways activated by Ras is the ERK pathway, and Ras-MEK signalling plays an important role in GLI1 regulation of human cancer cells (Lauth & Toftgard, 2007; Stecca et al, 2007; Varnat et al, 2009). We observed comparable levels of mammosphere inhibition by DN-Ras and the MEK inhibitor U0126 (Fig 7B). Expression of a constitutively active form of MEK increased mammosphere formation (Fig 7C), suggesting a role of Ras/MEK signalling in regulation of the GLI pathway. We also confirmed that BMI-1 was dependent upon GLI1 by expressing a dominant negative GLI1 in SUM1315 NRP2^high^ cells and observing reduced BMI-1 expression (Fig 7D). Similarly, GLI1 increased BMI-1 expression in the NRP2^low^ population of cells isolated from human breast tumours (Fig 7E). Moreover, exogenous GLI1 rescued BMI-1 from the inhibitory effect of dominant-negative Ras, suggesting that GLI1 is downstream of Ras (Fig 7F).

To establish the role of GLI1 in NRP2-mediated tumour initiation, we expressed GLI1 in the SUM1315 NRP2^low^ cells and assayed their ability to form orthotopic tumours. Expression of GLI1 decreased the incidence of tumour-free survival significantly demonstrating that GLI1 is sufficient to compensate for low NRP2 expression in tumour initiation (Fig 7G). Moreover, down-regulation of GLI1 or inhibition of NRP2 in SUM1315 NRP2^high^ cells significantly decreased tumour initiation (Fig 7H).

**Figure 8:**
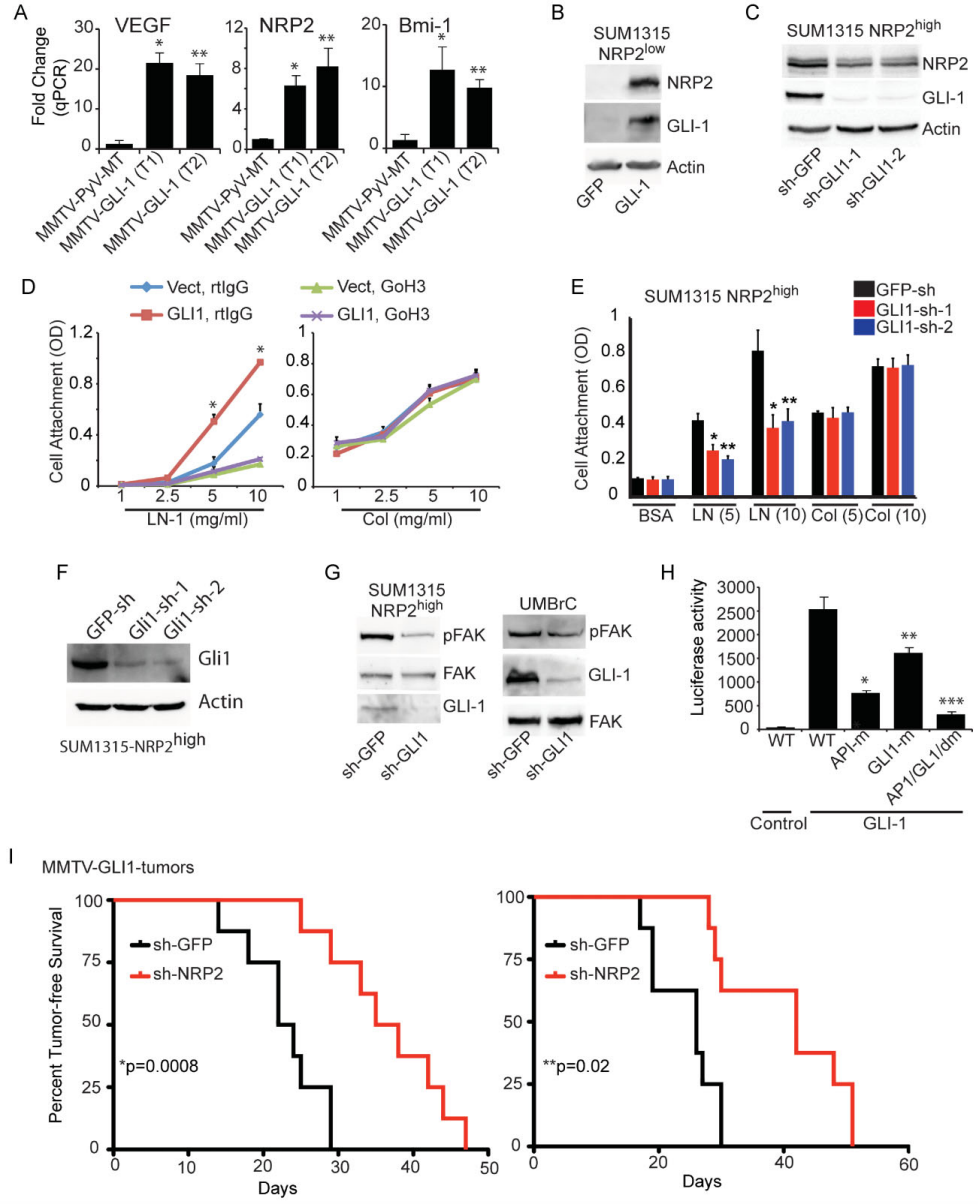
GLI1 stimulates an autocrine loop involving VEGF/NRP2 and the α6β1 integrin A. Mammary tumours isolated from transgenic mice that express either GLI1 or PyV-MT under the regulation of the MMTV promoter were analysed for expression of VEGF, NRP2 and BMI-1 by qPCR. *p = 0.002, **p = 0.006 (for VEGF); *p = 0.01, **p = 0.01 (for NRP2); *p = 0.029, **p = 0.001 (for BMI-1). B. A GLI1 construct was expressed in the NRP2^low^ population of SUM1315 cells and the effect on NRP2 expression was assayed by immunoblotting. C. GLI1 expression was depleted by shRNAs in the NRP2^high^ SUM1315 population and NRP2 expression was measured by immunoblotting (*p = 0.001). D. A GLI1 construct was expressed in the NRP2^low^ population of SUM1315 cells and the ability of these cells to adhere to laminin or collagen in the presence of an α6 blocking Ab or control IgG was assayed. E,F. GLI1 expression in the NRP2^high^ population of SUM1315 cells was depleted using shRNAs (GLI1-sh-1 and GLI1-sh-2), and the ability of these cells to adhere to either laminin or collagen (5 or 10 µg/ml) was assessed (E). *p = 0.003, **p = 0.002 (for LN, 5 µg/ml); *p = 0.008, **p = 0.01 (for LN, 10 µg/ml). Extracts from these populations were immunoblotted to verify GLI1 knockdown (F). G. GLI1 expression was depleted by shRNAs in the NRP2^high^ populations isolated from SUM1315 cells and human breast biopsies (UMBrC) and the effect on FAK expression and phosphorylation (pY397) was assessed by immunoblotting. H. A GLI1 construct was expressed in the NRP2^low^ population of SUM1315 cells. These transfectants were used to assay NRP2 promoter activity using luciferase reporter constructs that contained the wild-type promoter or mutations in the AP-1 and GLI1-binding sites. The above experiments were repeated at least twice (*p = 0.008; **p = 0.02; ***p = 0.004). I. Freshly isolated tumour cells from MMTV-GLI1 mice (two independent tumours) were infected with either GFP or NRP2 shRNAs (8 mice per group). These cells were implanted in the mammary fat pads of NSG mice and tumour formation was assessed by palpation. The curve comparison was done using the Log-rank test (*p = 0.0008; **p = 0.02). Error bars represent the mean ± SD for the *in vitro* experiments. Statistical differences between data groups were determined using Student's *t*-test.

More definitive data to evaluate the role of GLI1 in tumour initiation and its relationship to NRP2 were obtained using a transgenic mouse model of breast cancer in which GLI1 is expressed under the regulation of the MMTV promoter (Fiaschi et al, 2009). These GLI1-induced tumours exhibit expansion of a population of epithelial cells expressing the progenitor cell markers keratin 6 and BMI-1 (Fiaschi et al, 2009). Initially, we compared the expression of VEGF, NRP2 and BMI-1 in MMTV-GLI1 tumours with MMTV-PyMT tumours and observed dramatically higher expression of these molecules in the MMTV-GLI1 tumours (Fig 8A). The fact the expression of VEGF and NRP2 was elevated in response to GLI1 expression suggested the existence of an autocrine loop in which VEGF/NRP2 and α6β1 regulate GLI1 and GLI1 regulates their expression and function. To explore this hypothesis, we assessed whether GLI1 regulates NRP2 expression. Indeed, the data reveal regulation of NRP2 by GLI1 as evidenced by the induction of NRP2 in SUM1315 NRP2^low^ cells that express exogenous GLI1 (Fig 8B) and reduced NRP2 expression in response to GLI1 depletion in SUM1315 NRP2^high^ cells (Fig 8C).

The existence of an autocrine loop implies that GLI1 should impact the function of the α6β1 integrin and FAK activation. This possibility is supported by our finding that exogenous GLI1 expression in SUM1315 NRP2^low^ cells increased adhesion to laminin but not collagen and that this increased adhesion is inhibited by an α6 function-blocking Ab (Fig 8D). Similarly, depletion of GLI1 expression in SUM1315 NRP2^high^ cells reduced adhesion to laminin but not to collagen (Fig 8E–F). GLI1 depletion in SUM1315 NRP2^high^ cells and in the NRP2^high^ population sorted from breast tumour specimens reduced FAK activation (Fig 8G).

We found that NRP2 has a GLI1 binding consensus sequence in its promoter region, as well as an AP1 site that has been shown to be important for regulating NRP2 expression (Goel et al, 2012a). To evaluate the relative contribution of GLI1 and AP1 binding sites to NRP2 promoter activity, we generated luciferase reporter constructs in which either one or both of these sites were mutated. We observed significant inhibition of promoter activity when either site was mutated and a synergistic effect when both sites were mutated (Fig 8H).

Finally, we assessed the role of NRP2 in MMTV-GLI1 tumourigenesis. For this purpose, we used freshly harvested tumour cells from two MMTV-GLI1 transgenic mice and depleted NRP2 expression using shRNAs. The ability of these cells to form orthotopic tumours in the mammary fat pad was evaluated. A significant increase in tumour-free survival was observed in NRP2-depleted cells compared to control cells providing evidence for the importance of NRP2 in GLI1-mediated tumourigenesis (Fig 8I).

### NRP2 is a potential therapeutic target for triple-negative breast cancer

**Figure 9:**
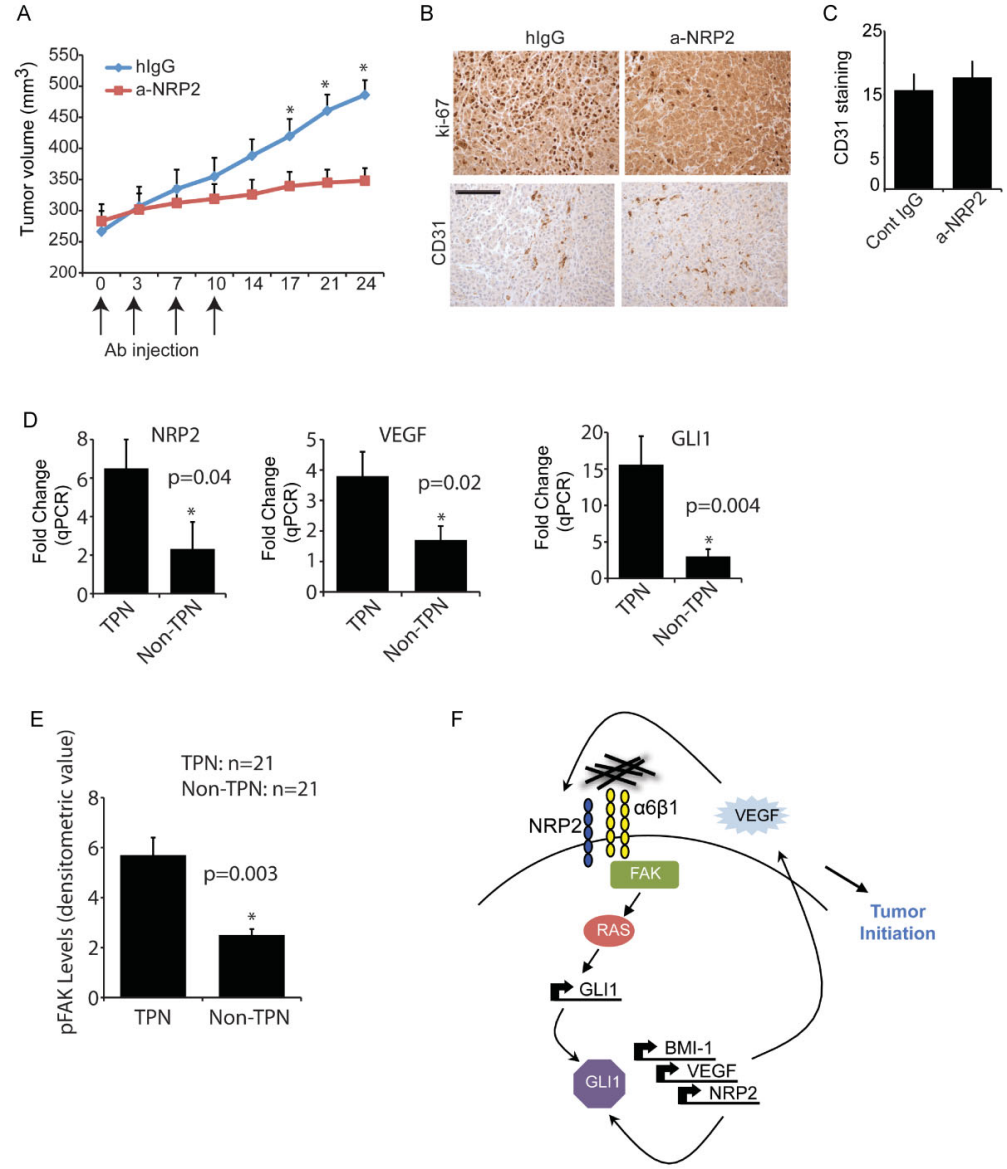
NRP2 inhibition impacts established tumours A. SUM1315 cells were implanted into mammary fat pads of NSG mice. Once tumours reached a volume of 250 mm^3^ as determined by caliper measurement, the mice were injected i.p. with either Anti-Nrp2^B^ or control IgG (twice weekly; 5 mice per group) and tumour volume was measured at regular intervals. Tumour growth was analysed by measuring tumour volume. Anti-Nrp2^B^ treatment resulted in a statistically significant inhibition of tumour volume. *p = 0.05 (day 17); *p = 0.008 (day 21); *p = 0.002 (day 24). B. Formalin-fixed and paraffin-embedded sections from SUM1315 tumours treated with either either Anti-Nrp2^B^ or control IgG (*n* = 3) were immunostained for either Ki-67 or CD31. Scale bar = 100 µm. C. CD31 staining was quantified to provide an indication of tumour angiogenesis. Quantification was done using 20 images from each group (*p = 0.5). D. Frozen clinical specimens from TPN (*n* = 32) or non-TPN (*n* = 35) breast tumours were used to compare the mRNA levels of NRP2, VEGF and GLI1 by qPCR. E. Frozen clinical specimens from TPN (*n* = 21) or non-TPN (*n* = 21) were used to compare the protein levels of pFAK by immunoblotting. A graph summarizing the densitometric analysis of the immunoblots is shown. F. Schematic model depicting the major conclusions of this study. Error bars represent the mean ± SD for all panels. Statistical differences between data groups were determined using Student's *t*-test.

Based on the findings that NRP2 plays an important role in tumour initiation, we evaluated the potential of a function-blocking NRP2 Ab (Anti-Nrp2^B^) to impact established tumours. Treatment of mice harboring orthotopic SUM1315 tumours with Anti-Nrp2^B^ reduced tumour size significantly (Fig 9A). This reduction in tumour size was associated with a reduced Ki-67 expression (Fig 9B). Surprisingly, NRP2 inhibition had no significant effect on angiogenesis as assessed by CD31 staining (Fig 9B and C). To validate the existence of the proposed pathway in clinical specimens, we quantified the expression of VEGF, NRP2 and GLI1 in 32 TPN and 35 non-TPN human breast tumour specimens by qPCR. The results obtained reveal that triple-negative breast tumours express significantly more of these molecules than non-triple-negative breast tumours (Fig 9D). Also, we analysed the expression of phospho-FAK by immunoblotting extracts from 21 triple-negative and 21 non-triple-negative breast tumours. The densitometric analysis of these immunoblots is provided in Fig 9E and it demonstrates that triple-negative breast tumours express significantly more phospho-FAK than non-triple-negative breast tumours.

Collectively, the data presented reveal a novel autocrine signalling pathway that contributes to the initiation of TNBCs that is amenable to therapeutic intervention. This pathway is mediated by the surface receptors VEGF/NRP2 and α6β1 integrin, which activate FAK/Ras signalling culminating in the enhanced expression of GLI1 and the consequent induction of BMI-1 (Fig 9F).

## DISCUSSION

This study reveals a novel autocrine signalling pathway involving VEGF/NRP2 and the α6β1 integrin that contributes to the initiation of TNBC. Although other studies have demonstrated the importance of autocrine VEGF/NRP signalling in tumour initiation (Beck et al, 2011; Hamerlik et al, 2012), no study to date had defined a mechanism that links this signalling to tumour initiation. The nexus of the pathway we define is the Hh target GLI1, which is regulated by concerted VEGF/NRP2 and α6β1 signalling. Importantly, GLI1 can enhance NRP2 expression and the function of the α6β1 integrin, establishing this autocrine pathway. A critical feature of GLI1 in this context is its ability to induce BMI-1, a transcriptional repressor that has been implicated in the function of mammary tumour stem cells (Glinsky et al, 2005; Liu et al, 2006). Given that NRP2 can be targeted effectively *in vivo* by Ab inhibition (Caunt et al, 2008), our findings support the feasibility of NRP2-based therapy for TNBC because such therapy would dismantle this autocrine loop and impede the function of TICs.

Arguably, the identification and characterization of cells that have tumour initiating potential is one the most significant problems in advancing our understanding of breast and other cancers and improving therapy. TNBC has been reported to harbour a higher frequency of such TICs than many other breast cancer sub-types (Idowu et al, 2012; Park et al, 2010; Polyak & Weinberg, 2009) consistent with the hypothesis the frequency of TICs increases with tumour grade and that such cells are associated with metastatic potential (Park et al, 2010; Pece et al, 2010; Polyak & Weinberg, 2009). To date, several studies have identified key signalling pathways that contribute to the function of breast cancer stem or TICs (DiMeo et al, 2009; Fillmore et al, 2010; Ginestier et al, 2010; Kim et al, 2012a; Korkaya et al, 2011; Marotta et al, 2011; Polyak & Weinberg, 2009; Sansone et al, 2007; Scheel et al, 2011). A strength of our study is that we link an autocrine signalling pathway involved in tumour initiation directly to the regulation of a key stem cell factor (BMI-1) that has been implicated in self-renewal (Liu et al, 2006) and in the EMT (Song et al, 2009). Moreover, BMI-1 expression has been associated with TNBC (Wang et al, 2012). These observations, therefore, are consistent with the fact that TNBC exhibits EMT characteristics (Jeong et al, 2012) and that VEGF/NRP signalling can induce an EMT (Mak et al, 2010).

Our data implicate NRP2 as a VEGF receptor that has a critical role in the initiation of TNBC. More specifically, we show that NRP2 has a causal role in the ability of breast cancer cells to form mammospheres *in vitro* and initiate tumours *in vivo*. A critical result supporting this conclusion is that NRP2 inhibition delayed the onset of tumours in a mouse model of TNBC (Fig 2H). Interestingly, other recent studies have highlighted the importance of autocrine VEGF/NPR1 signalling in the initiation of skin tumours and glioblastomas (Beck et al, 2011; Hamerlik et al, 2012). Although our study affirms the importance of autocrine VEGF/NRP signalling in tumour initiation, it is the first such study to implicate NRP2.

Our demonstration that the α6β1 integrin functions in concert with NRP2 to drive autocrine VEGF signalling is significant because this integrin has been implicated in the function of tumour stem cells (Lathia et al, 2010) and high α6 expression characterizes tumour initiating populations (Friedrichs et al, 1995; Honeth et al, 2008; Lathia et al, 2010; Mulholland et al, 2009; Schober & Fuchs, 2011; Vieira et al, 2012). However, the mechanism by which it functions in this context has been elusive. We conclude that the ability of this integrin to activate FAK and the consequent activation of Ras and induction of GLI1 expression underlie its contribution to tumour initiation. Indeed, our observation that an integrin involved in tumour initiation regulates a Hh effector molecule that is also involved in tumour initiation is novel and unexpected. The α6β1 integrin functions primarily as a laminin receptor (Goel et al, 2012b), and this function is supported by our finding that GLI1 expression induces cell adhesion to laminin but not collagen and this induction is dependent on α6β1. These observations support the conclusion that laminin is an important component of the matrix microenvironment that regulates the function of TICs and that these interactions are mediated by α6β1 (Lathia et al, 2010). Our data add a new dimension to the understanding of the role of α6β1 in tumour initiation because they indicate that the function of this integrin is regulated by VEGF/NRP2 signalling consistent with our previous finding that NRP2 interacts specifically with α6β1 and facilitates its association with the cytoskeleton and localization in focal adhesions (Goel et al, 2012b).

Recent studies have demonstrated the importance of FAK in breast cancer and the function of TICs (Ginestier et al, 2010; Luo et al, 2009). For example, targeted deletion of FAK inhibited tumourigenesis in a mouse model of mammary carcinoma and reduced the pool of cancer stem/progenitor cells in these tumours (Luo et al, 2009). The question that arises from these seminal studies is how FAK contributes to these functions. Our data reveal that FAK is essential because it regulates the expression of BMI-1 by activating Ras and inducing GLI1 expression. The involvement of FAK in regulating BMI-1 expression does not exclude other contributions of this kinase to the function of TICs, although its ability to induce GLI1 and, consequently, BMI-1, provides an important mechanism for its involvement in tumour formation.

The culmination of our study is the discovery that GLI1 functions at the nexus of the autocrine VEGF/NRP2 and α6β1 signalling pathway that contributes to the initiation of TNBC. This function of GLI1 is substantiated by our finding that the expression of VEGF, NRP2 and GLI1 is significantly higher in TNBC than in non-TNBC (Fig 9D). Other studies have also implicated GLI1 in the function of tumour stem or initiating cells (Liu et al, 2006; Varnat et al, 2009). The fact GLI1 can regulate BMI-1 expression (Liu et al, 2006) also strengthens its involvement in the function of TICs. As mentioned, the ability of the α6β1 integrin to regulate GLI1 expression was unexpected, as was our finding that GLI1 regulates the function of α6β1 and its ability to function as a laminin receptor. We postulate that the regulation of α6β1 function by GLI1 is mediated by GLI1 regulation of NRP2. This conclusion is supported by the reports that NRPs are positive regulators of Hh signalling (Cao et al, 2008; Hillman et al, 2011). Previously, we established the importance of c-Jun in regulating NRP2 transcription (Goel et al, 2012a) and we demonstrate here that c-Jun and GLI1 function synergistically to regulate NRP2. Interestingly, GLI1 can regulate c-Jun (Laner-Plamberger et al, 2009) suggesting that the positive feedback inherent in our autocrine signalling pathway would enhance both GLI1 and c-Jun, and impact NRP2 and α6β1.

An important issue that emerges from our findings is whether canonical or non-canonical Hh signalling contributes to the signalling pathway we describe. The data we present strongly suggest the involvement of a non-canonical pathway because down-regulation of Smoothened, a critical component of the canonical pathway, did not inhibit autocrine signalling. This observation is consistent with our conclusion that FAK-mediated Ras activation drives GLI1 expression and it supports previous studies that have implicated Ras in non-canonical Hh signalling (Lauth & Toftgard, 2007; Stecca et al, 2007; Varnat et al, 2009). Yet, we also found that exogenous Shh increased mammosphere formation (Fig 6B). One interpretation of these findings is that non-canonical GLI1 activation can be amplified by canonical signalling. This possibility is supported by the reports that simultaneous activation of Ras and Hh signalling caused extensive formation of intraepithelial neoplasias and accelerated lethality in a transgenic model of pancreatic carcinoma (Pasca di Magliano et al, 2006) and that de-regulation of Ras signalling in mice lacking p53 increased Shh expression (Hingorani et al, 2005). Moreover, our finding that down-regulation of Smoothened reduced FAK-mediated up-regulation of GLI1 (Fig 6D) supports this hypothesis.

Our conclusion that GLI1 is induced by a NRP2-mediated Ras pathway should be discussed in the context of the existing literature on how Ras mediates GLI1 activation. Ras can regulate the nuclear localization of GLI1 via SUFU based on the report that enhanced levels of SUFU prevented nuclear localization of GLI induced by oncogenic Ras (Stecca et al, 2007). However, SUFU does not appear to have a major role in NRP2-mediated GLI1 activation based on our data. Our results do agree with the finding that Ras mediates GLI1 activity via the MEK/ERK pathway and they implicate NRP2 as a mediator of this pathway. Although there is evidence that p53 inhibits the activity and nuclear translocation of GLI1 (Stecca and Ruiz i Altaba, 2009), this mode of GLI1 regulation is probably not relevant for our findings because TNBC is predominantly p53 mutant (Kumar et al, 2012; Network, 2012).

Finally, the data presented have significant implications for therapy of breast cancer, especially TNBC (Caunt et al, 2008). Specifically, targeting NRP2 could be effective in reducing tumour burden and recurrence because it will disrupt an autocrine signalling pathway that is necessary for the function of TICs, in addition to inhibiting lymphangiogenesis as previously suggested (Caunt et al, 2008). Indeed, our finding that NRP2 Ab treatment of tumours reduced tumour volume but it did not inhibit tumour angiogenesis significantly suggests that anti-NRP2 therapy could be highly effective when used in conjunction with anti-angiogenesis therapies for the clinical management of breast cancer.

## MATERIALS AND METHODS

### Reagents and antibodies

Matrigel and collagen I were purchased from BD Biosciences (San Jose, CA, USA) and laminin-1 (LN-1 or LN-111) was purchased from Invitrogen (Carlsbad, CA, USA); PF573228 (Tocris). The following Abs were used: α6 (AA6A, provided by Anne Cress; and GoH3, purchased from Millipore); NRP2 (goat IgG, R&D; C9 and H300, Santa Cruz, Santa Cruz, CA, USA); actin (Sigma); p-FAK Y397 (mouse IgG, BD Bioscience) and FAK (Santa Cruz); EpCAM (AbCaM); Flag (Sigma); ki-67 (NovoCastra); HA (Roche); CD31 (Santacruz); p-ERK, ERK and GLI1 (Cell Signaling); BMI-1 (Cell Signaling) and VEGF (Calbiochem). A function-blocking NRP2 Ab (Anti-Nrp2^B^) was provided by Genentech (Caunt et al, 2008).

### Isolation of epithelial cells from breast tumours

Human breast tissue was obtained in compliance with the Institutional Review Board of the University of Massachusetts Medical School. Epithelial cells were isolated from discarded but freshly resected, invasive breast tumours as described (Fillmore et al, 2010). Briefly, the tissue was minced and digested overnight with a mixture of collagenase (Roche, Indianapolis, IN, USA) and hyaluronidase (MP Biomedicals, Solon, OH, USA). The digested cells were plated briefly in serum (1–2 h) to deplete mammary fibroblasts. The organoids were dissociated into a single cell suspension by trypsinization and filtered (40-µm filter; BD Biosciences) to remove residual clustered cells. Immediately after dissociation, cells were sorted based on EpCAM, α6 and NRP2 expression, and subsequently analysed by either immunoblotting or FACS. Ascites fluid was collected from breast cancer patients (5094M and 5104M) with metastatic disease. Malignant cells were pelleted by centrifuging ascitic fluid diluted with PBS. The cell pellet was washed several times with PBS and tumour cells were separated using Ficoll. 5094M is ER^+^/PR^−^ and 5104M is ER^+^/PR^+^/Her2^−^.

**The paper explained**

**PROBLEM**

Highly aggressive forms of breast carcinoma are difficult to treat because they progress rapidly and are resistant to most chemotherapeutic drugs. The reason for their aggressive behaviour and drug resistance is that these tumours contain a high number of cells that have the ability to form new tumours, cells that are referred to as tumour initiating or stem cells. These cells also have the propensity to metastasize. For these reasons, TICs are ideal targets for therapy but much more needs to be known about the mechanisms that contribute to their function.

**RESULTS**

We establish that TICs present in highly aggressive breast carcinomas require autocrine VEGF signalling mediated by the receptor neuropilin-2 (NRP2) for their function. Furthermore, we define a signalling cascade initiated by VEGF/NRP2 signalling that culminates in the regulation of a key stem cell factor BMI-1 that is critical for tumour initiation. This signalling cascade involves the ability of VEGF/NRP2 signalling to activate the a6b1 integrin and, consequently, FAK. FAK-mediated activation of Ras/MEK signalling enhances expression of the Hedgehog effector GLI1 that can induce BMI-1. Importantly, GLI1 also stimulates NRP2 expression, establishing a positive feedback loop that sustains this autocrine pathway. The significance of these data is evidenced by the fact that inhibition of NRP2 prevents tumour initiation and it causes regression of established tumours.

**IMPACT**

The data presented provide a novel mechanism that contributes to the initiation of aggressive breast carcinomas. This mechanism integrates the function of several key molecules that have been implicated in the function of tumour stem cells. Importantly, these findings provide a strong rationale for the use of anti-NRP2 therapy for the treatment of aggressive breast carcinomas.

### Cell lines

SUM1315, SUM149 and SUM159 cells were provided by Dr. Steve Ethier (Wayne State University School of Medicine). MCF-10A and MCF-10AT were obtained from the Barbara Ann Karmanos Cancer Institute (Detroit, MI, USA). HMECs were purchased from Lonza Bioscience and HMLE-PR cells were provided by Dr. Weinberg (Whitehead Institute).

### Molecular reagents

Lentiviruses containing shRNAs specific for NRP2 (clone ID TRCN0000063308, TRCN0000063309 or TRCN0000063312), α6 integrin (TRCN0000057777, TRCN0000057776); GLI1 (TRCN0000020484, TRCN0000020488); Smoothened (TRCN0000014366 and TRCN0000014367) or GFP (RHS4459) were obtained from Open Biosystems (Huntsville, AL, USA). A lentiviral plasmid (FUGW) expressing BMI-1 was obtained from Addgene. A constitutively active FAK construct was provided by Jun-Lin Guan (University of Michigan Medical School). Wild-type and dominant negative Ras constructs were described previously (Yoon et al, 2006). Wild-type GLI1 and Gli3T (dominant negative GLI1, (Rajurkar et al, 2012)) constructs were provided by Dr. Junhao Mao. A NRP2 plasmid was obtained from Dr. Tsai (Baylor College of Medicine) and mutations in the b1 and b2 domains of NRP2 were made in this plasmid using SLIC cloning (Li & Elledge, 2007). A NRP2 promoter luciferase construct (−3000/+195) or the same construct with AP1 mutations was described previously (Goel et al, 2012a). A mutation in the GLI1 binding site (ACCACCCA to AAAAAAAA) in these constructs was generated using Quickchange XL Site-directed Mutagenesis kit (Agilent Technologies). A construct expressing constitutively active Ras was obtained from Addgene.

### Cell-based assays

Cell adhesion assays were performed as described previously (Goel et al, 2012b). Flow cytometry was used to quantify surface expression of the α6 integrin and NRP2 in SUM1315 and UMBrC cells. The gating strategy for isolating luminal (CD44^+^/CD24^+^/EpCam^+^) and stem-like (CD44^+^/CD24^−^/EpCAM^+^) populations in Fig. 1 was described previously (Gupta et al, 2011).

To generate mammospheres, single-cell suspensions were maintained in DMEM-F12 medium supplemented with B27 (1:50; Invitrogen), EGF (20 ng/ml), basic fibroblast growth factor (20 ng/ml;bFGF), and heparin (10 µg/ml) in Costar 3471 six-well plates at a density of 2 × 10^4^ cells/ml. In some experiments, mammospheres were collected by centrifugation and dissociated with 0.05% trypsin for 15 min to obtain single-cell suspensions (Liao et al, 2007).

### Immunoblotting and qPCR

Cells were extracted in either a Triton-X-100 buffer (1% Triton X-100, 150 mM NaCl, 50 mM Tris–HCl, pH 7.5, 1 mM PMSF and protease inhibtors) or RIPA (50 mM Tris–HCl, pH 7.4, 150 mM NaCl, 0.1% SDS, 0.5% sodium deoxycholate, 1 mM PMSF and protease inhibitors). The proteins were separated by SDS–PAGE and immunoblotted using Abs as specified in the figure legends. For qPCR, RNA was isolated using RNeasy kit (Qiagen) and converted to cDNA using Transcriptor First Strand cDNA Synthesis kit (Roche). We performed qPCR with a SYBR Green PCR master mix using an ABI Prism 7900HT instrument (Applied Biosystems).

### Xenograft experiments

SUM1315 cells were mixed with Matrigel at a 10-fold dilution and injected into a mammary fat pad of immunocompromised NOD.Cg-*Prkdc*^*scid*^ *IL2rg*^*tm1Wjl*^ (abbreviated as NSG) mice. Tumour onset was determined by palpation. In some experiments, SUM1315 xenografts were treated intraperitoneally (i.p.) with either control IgG or a NRP2 Ab (Anti-Nrp2^B^) at dose of 40 mg/kg (twice weekly). In some experiments, NRP2^low^ population of SUM1315 cells expressing either a control vector or GLI1 construct were injected in the mammary fat pad of NSG mice and tumour onset was determined. To study the role of NRP2 in MMTV-Gli tumours, primary tumour cells from MMTV-GLI1 mammary tumours were infected with either NRP2 or GFP shRNAs and injected orthotopically in NSG mice. Tumour onset was used as the endpoint for these studies.

### Transgenic mice

MMTV-PyV-MT mice transgenic for the polyoma virus middle T (PyV-MT) antigen under the control of the MMTV promoter (Jackson Laboratory) were obtained from Dr. Leslie Shaw. Cells from these mice are isolated and maintained as described before (Nagle et al, 2004).

The TgMFT121; Brca1f/f p53f/f; TgWAP-Cre (TBP) model was generated and described before (Kumar et al, 2012). In brief, TgMFT121 (MMTV-Floxed-eGFP-T121) were generated to conditionally inactivate the pRb family of proteins in mammary epithelium. TgMFT121 mice were crossed with TgWAp-Cre, Brca1f/f and p53f/f (NCI Mouse Repository Frederick) to generate female TgMFT121; Brca1f/f p53f/f; TgWAP-Cre mice, this approach inactivate pRB family proteins, Brca1 and p53 specifically in mammary epithelial cells via the Wap-Cre transgene. All mouse strains used to generate TBP mice were backcrossed to FVB background for 10 generations.

The MMTV-GLI1 transgenic mouse model was generated and described before (Fiaschi et al, 2009). These mice express transgenic GLI1 in the presence of doxycycline, which was added to drinking water with 5% sucrose. All animal experiments were in accordance with institutional guidelines and are approved by IACUC.

## Author contributions

HLG and AMM designed experiments and interpreted data; HLG, BP and CC carried out *in vitro* cell experiments; HLG, BP and JHN were involved with *in vivo* experiments on mouse models; JM assist in GLI1 *in vitro* experiments; CWVK was involved in peptide design and synthesis. LMS, RT, PK and KS were involved *in vivo* transgenic mouse models; LDS and DLG were involved in xenograft experiments; HLG, CK and AMM wrote the manuscript. HLG and AMM carried out the data analysis.

## Notes

As a service to our authors and readers, this journal provides supporting information supplied by the authors. Such materials are peer reviewed and may be re-organized for online delivery, but are not copy-edited or typeset. Technical support issues arising from supporting information (other than missing files) should be addressed to the authors.
