# Supplementary material for: GLI1 regulates a novel neuropilin-2/α6β1 integrin based autocrine pathway that contributes to breast cancer initiation
Source: EMBO Mol Med. 2013 Feb 21;5(4):488–508. doi: 10.1002/emmm.201202078 (PMC3628099; doi:10.1002/emmm.201202078)
Supplement: Supplementary file 1 [file emmm0005-0488-sd1.pdf]

Gli1 regulates a novel Neuropilin-2/alpha6beta1 integrin based autocrine pathway that contributes to breast cancer initiation

Hira Lal Goel, Bryan Pursell, Cheng Chang, Leslie M. Shaw, Junhao Mao, Karl Simin, Prashant Kumar, Craig W. Vander Kooi, Leonard D. Shultz, Dale L. Greiner, Jens Henrik Norum, Rune Toftgard Charlotte Kuperwasser and Arthur M. Mercurio

Corresponding author: Hira Lal GOEL, University of Massachusetts Medical School

Review timeline:

Submission date:	25 September 2012
Editorial Decision:	06 November 2012
Revision received:	10 January 2013
Editorial Decision:	15 January 2013
Revision received:	15 January 2013
Accepted:	16 January 2013

Transaction Report:

(Note: With the exception of the correction of typographical or spelling errors that could be a source of ambiguity, letters and reports are not edited. The original formatting of letters and referee reports may not be reflected in this compilation.)

Editor: Roberto Buccione

1st Editorial Decision 06 November 2012

Thank you for the submission of your manuscript to EMBO Molecular Medicine. We have now heard back from the three referees whom we asked to evaluate your manuscript.

Although the referees find the topic of the study interesting and important, they also raise a number of substantial concerns about the conclusiveness and completeness of the results and on technical issues, which should be convincingly addressed in a major revision of the current manuscript.

Reviewer 1 raises doubts on the overall novelty of your findings but does acknowledge the potential relevance of the new autocrine pathway proposed. However, according to the Reviewer, critical experiments to support this claim are missing. S/he also expresses concern on the heterogeneity of cell lines used in the different assays.

Reviewer 2, while acknowledging the potential impact of your work, does raise substantial concerns with respect to its translational potential. S/he suggests that a more appropriate breast cancer model should be used and maintains that the role of Gli2 should be analysed and that the role of Ras has not been appropriately addressed (see also Reviewer 1). Finally, the Reviewer asks whether the components of the NRP2-VEGF-FAK-Gli1 axis are actually expressed in triple negative tumours. This data would considerably strengthen the conclusions of the study. Reviewer 2 would also like to see which factors bind to the Gli1 promoter to control its expression downstream of NRP2; I do appreciate, however, that this is a further reaching request and would imply considerable work. I

would be satisfied if all other concerns are carefully dealt with.

Finally, I would also like to draw your attention to the numerous and strong concerns on wording, appropriateness of citations and the excessive slant towards cancer stem cells, expressed by Reviewers 1 and 3. These criticisms should be scrupulously addressed.

While it is clear that publication of the paper cannot be considered at this stage, I am open to the submission of a revised manuscript providing that the Reviewers' concerns are fully addressed.

I should remind you that it is EMBO Molecular Medicine policy to allow a single round of revision only and that, therefore, acceptance or rejection of the manuscript will depend on the completeness of your responses included in the next, final version of the manuscript.

As you know, EMBO Molecular Medicine has a "scooping protection" policy, whereby similar findings that are published by others during review or revision are not a criterion for rejection. However, I do ask you to get in touch with us after three months if you have not completed your revision, to update us on the status.

I look forward to seeing a revised form of your manuscript.

***** Reviewer's comments *****

Referee #1 (General Remarks):

The paper by Goel et al. reports the effects of blockade of Neuropilin-2 in triple negative breast cancer cells in vitro and in vivo.

Overall this is interesting but not entirely novel.

Mechanistically, the authors report interesting effects of FAK, alpha6beta1 integrin and Gli1 in these cells. Most of the data is well presented and results clear. However, there is a general lack of novelty in each specific area given the many papers previously published on the different parts. For instance on nrp2 in cancer, nrp2 on HH signaling, integrins and nrp2 on cancer stem cells, Gli and Bmi1, Bmi1 and mmamospheres, Gli and RAS, etc.

This paper attempts to brig it all together and propose a novel autocrine pathway but the ends are not tight and critical experiments are missing.

Specific points.

1-There is a general lack of precision on the effects of kd by shRNAs in vitro and in vivo. The levels of kd are not given and so it is not clear if the small effects of shNRP2 on tumor growth (Fig. 2G) are due to inefficient kd or not.

2-What is the role of endogenous NRP2 in mice? Does the Ab block this as well as the human form? Why are mice not affected in Fig. 2H?

3-Where is the proof of the specificity of the NRP-2 Ab? Can the authors provide rescue experiments in vivo without enhancing high GLI1 levels, for example by kd of SUFU as they suggest in the discussion?

4- Fig. 5B suggests inhibition of GLI1 protein function in a reporter assay by kd of NRP2. How is this thought to happen?

5- The analyses with shSMO are interesting but a critical control is missing. Does shSMO inhibit GLI1? If so, it should inhibit the autocrine loop and block growth. Why does it not have this effect? Does shSMO inhibit mammosphere formation?

6- Conclusions with gain-of-function of GLI1 only can be tricky as this is a potent oncogene. The

fact that FAK increases GLI1 expression does not mean that GLI1 mediates FAK function. More careful writing is needed.

7-The section of RAS is misguided and misreferenced. Lauth and Toftgard is a review that does not directly address RAS work. Primary previous work has shown the GLI regulalton by RAS, AKT and MEK (for instance Steca et al., 2007; Varnat et al., 2009). Guan et al is not related to this topic. These papers have shown GLI1 is downstream of RAS in human cancer cells of various types.

8- It is not clear what is the level of GLI1 expressed in SUM1315 NRP2low cells. Too high levels can be toxic or induce a dominant oncogenic response that may not be related to epistatic effects. Again, performing such experiments with shSUFU or blockade of other endogenous factors that activate GLi1 may be more informative.

9-Why does overexpression of GLI1 lead to reduced adhesion to laminin but not collagen (Fig 7E-F_?

10- Is is important to resolve whether Gli1 is required for SUM1315 tumor growth in vivo. Is kd of GLI1 synergistic with the effects of the anti-NRP2 Ab?

11- If NRP2 is causal for mammosphere formation and mammosphere cells cause tumor initiation, why does blockade of NRP2 only delay tumor growth in vivo (p.14)?

12- What is the proof that GLI1 is RAS-dependent in vivo?

13- The localization of GLI1 protein by Xu et al is largely unreliable.

13- Additional references are required for the point of the role of Gli1 in cancer initiating cells (e.g. Clement et al., 2007; Peacock et al., 2007) and that Shh increases clonogenicity.

14- The mechanistic speculation needs a lot of work (p.15,16) incorporating previous works on GLI, ras, sufuh in multiple systems (e.g. Stecca et al., 2007; Varnat et al, 2009) and p53 (Abe et al., 2008; Stecca and Ruiz i Altaba, 2009). Care should also be taken equating mouse and human cell behavior.

15- It is also necessary to use the same cells in all assays. At this point it is not clear why are so many different cells used in the different assays.

Overall, this is a study that has lots of interesting parts, but most are not truly novel. Those more novel ones are, unfortunately, not sufficiently developed.

Referee #2 (General Remarks):

The study by Goel et al describes a signaling pathway initiated by the NRP2 receptor leading to the activation of the transcription factor GLI1 and increase levels of breast cancer initiating cells. Although the study has some overlap with recently published reports describing the regulation of GLI1 by NRP1 and NRP2, it has great biological and translational relevance to the field of breast carcinogenesis. The authors have extensively characterized this NRP2-GLI1 pathway and its impact in tumor initiation cells using well-established in vitro and in vivo assays. The data is clearly presented and the manuscript is well written. However, there are few aspects of the study that need additional experimentation to fully support the authors' conclusion. For instance, the therapeutic testing of the blockade of the pathways should be done in a relevant breast cancer model, the xenograft model lacks some biological features of triple negative tumors that may impact in the treatment's outcome. The authors should perform a set of experiments in the TBP model and define the targeting specificity by looking the activation of downstream molecules of NRP2 and expression of GLI1 and Bmi1. The mechanism controlling GLI1 should be further expanded, especially when the cell model used in the study are Hedgehog responsive. Is GLI2 involved in this regulatory mechanism? What are the factors binding to the GLI1 promoter to control its expression downstream of the NRP2 receptor? Results from these experiments will help future efforts to develop NRP targeted therapies. In addition, the role of Ras in this process should be further

validated, the dominant negative experiment is not ideal to define the role of this GTPase in the regulation of GLI1. Finally, the expression of this newly identified pathway in triple negative breast cancer should be examined in clinical specimens. Are the components of the NRP2-VEGF-FAK-GLI1 expressed in triple negative tumors?

Referee #3 (Comments on Novelty/Model System):

This is a very thorough manuscript establishing a novel autocrine loop that drives breast tumour cell growth. The author has used a variety of model systems, including cell lines, primary human breast tumours and mouse models.

Referee #3 (General Remarks):

The manuscript by Goel et al. describes the presence of an NRP2/Alpha 6 integrin-FAK-RAS-GLI1-VEGF autocrine loop that drives tumour cell growth in triple negative breast cancer. The manuscript is well written and presented, and the experiments describing the components of this autocrine mechanism are thorough. The manuscript is also strengthened by incorporating the use of mouse models and primary human breast tumour tissue into their experiments, and not just relying on the use of cell lines.

My concern regarding this manuscript is that there is a very strong slant towards cancer stem cells and promoting this autocrine mechanism as a cancer stem cell-specific mechanism. The manuscript is also plagued by some blanket statements regarding cancer stem cells that are simply not accurate. Most of the work describing breast cancer stem cells have relied on cell lines and the use of mammosphere assays - hardly a robust model system. A recent publication has challenged the use of sphere assays as a good indicator of in vivo tumourigenicity (see: Kim et al (2012) PNAS 109:6124-9). Likewise, in vivo transplantation assays are problematic since it has been demonstrated that normal luminal stem cells do not engraft efficiently in the absence of appropriate basal helper cells (Van Keymeulen et al (2011) Nature) - this latter observation could have major implications for the detection of cancer stem cells that have a luminal phenotype. My advise to the authors is to tone down the cancer stem cell aspect and steer clear of this controversy, because otherwise it will mar an otherwise pretty good manuscript.

Comments
Page 3: "A distinguishing feature of TICs is their self-sufficiency and their use of autocrine and paracrine signaling pathways to sustain their function (DiMeo et al, 2009; Fillmore et al, 2010; Ginestier et al, 2010; Kim et al, 2012; Korkaya et al, 2011; Marotta et al, 2011; Sansone et al, 2007; Scheel et al, 2011)." This is a rather strange comment since I would expect that many different cell types in the body rely on autocrine/paracrine signalling for their functions and I hardly think that this is a property that distinguishes stem cells.

When sorting out the 6high population from primary human tissue, are these 6high cells also gated on an epithelial marker such as EpCAM? My worry is that if it is just alpha 6 alone, could you not also be enriching for endothelial cells? Endothelial cells also express alpha 6 integrin, and presumably would have high levels of NRP2 expression.

Page 6: "The 6high population, which expressed high NRP2 (Fig. 1D), formed mammospheres significantly more than the 6low population (Supporting Information Fig S1A)." Are the data in Fig S1A significantly different? If so, an asterisk should indicate this, or the wording changed.

Figure 1D dot plots: What is the background (fluorescence minus one or isotype control Ab) level of fluorescence these dot plots? This should be indicated.

Figures 5E: This is a very dramatic difference in the expression of Gli, Bmi1 and NRP2 between adherent cultures and sphere cultures (10-40X). How is this related to phenotype? Are adherent cells more luminal like? Are the sphere cells more basal/EMT like?

Page 3, first paragraph: "The hypothesis that breast tumors harbor a population of cells that has

stem-like properties and can initiate tumorigenesis is supported by strong experimental evidence (Baccelli & Trumpp, 2012; Gupta et al, 2009; Keller et al, 2012; Korkaya et al, 2011)." I do not agree that Keller et al is an appropriate reference for the presence of breast tumour stem cells - this manuscript was about the cellular origins of different types of breast cancer. Al-Hajj et al would be a better reference.

Page 3, first paragraph: "The frequency of such tumor initiating cells (TICs) is high in poorly differentiated tumors (Pece et al, 2010) and these cells may be intimately associated with an epithelial mesenchymal transition (EMT) and contribute to metastasis (DiMeo et al, 2009; Mani et al, 2008; Scheel et al, 2011)." The statement regarding the relationship between stemness and EMT (basal cell phenotype) is not always the case. See: Kim J et al. Proc Natl Acad Sci U S A. 2012 Apr 17;109(16):6124-9.

Figure 1A: Please show a flow dot plot showing how these luminal and stem cell populations were gated and sorted. Also, I would recommend that you call cell population not "luminal" or "stem", but instead label them according to their phenotype (e.g., CD44+/CD24-). It may be that the luminal cells have stem cell activity (see: Kim et al (2012) PNAS 109:6124-9), but you have not used the correct assay to detect this.

Graph in S1D: Is there not a significant difference between these two conditions? If so, an asterisk should indicate this.

Page 7: "We also established two cell lines from the ascites fluid of breast cancer patients. Both of these cell lines are able to form tumors in vivo (3 out of 5 mice after 135 days)." A sentence describing these tumours would be helpful (e.g., ER? HER2?). I would suggest deleting the 3 out of 5 mice and just state that these cell lines were tumourigenic in vivo. The frequency of tumour formation will be dependent on the cell dose and the conditions of the transplant (e.g., recipient mouse strain, inclusion of Matrigel in the transplant inoculumn.)

Page 7: "These tumors, in marked contrast to MMTV-PyV-MT tumors, express abundant NRP2, which is localized on the surface of tumor cells (Fig. 2E)." How can you tell this from the image presented in Figure 2E? You would have to establish cell surface expression by flow cytometry, not by IHC.

Page 9: "These findings are of particular interest since 6 1 (CD49f) is an established marker of many tumor stem-like cells including those of the breast (Lathia et al, 2010), and high expression of this integrin is a characteristic of TICs (Friedrichs et al, 1995; Honeth et al, 2008; Lathia et al, 2010; Mulholland et al, 2009; Schober & Fuchs, 2011; Vieira et al, 2012)." Need to be careful about making blanket statements. Many cells in the mammary gland express C49f (actually, most cells except the mature luminal cells in the normal breast express this protein), but not all of these cells are necessarily stem cells. Although stem cells express this marker, it is not a stem cell-specific marker.

Materials and Methods: qRT-PCR methods appear to be missing.

Figure 6A: This is relative fold-change...but to what?

1st Revision - authors' response 10 January 2013

Response to Reviewers' Comments

EMM-2012-02078

We thank the reviewers for their insightful and constructive comments. All of the concerns raised have been addressed and the revised manuscript contains a significant number of new *in vitro* and *in vivo* experiments, as well as an extensive analysis of human tumour specimens.

Referee #1

General Remarks

"*Most of the data is well presented and results clear. However, there is a general lack of novelty*".

We respectfully disagree with the reviewer's concern regarding novelty. The main focus of this manuscript is the characterization of novel autocrine loop mediated by VEGF/NRP2 that functions in the initiation of triple-negative breast tumours. This aspect of the study is novel and has significant implications for breast cancer as noted by the other reviewers.

Specific points.

1. "*There is a general lack of precision on the effects of kd by shRNAs in vitro and in vivo. The levels of kd are not given and so it is not clear if the small effects of shNRP2 on tumour growth (Fig. 2G) are due to inefficient kd or not*"

In response, we have provided an immunoblot blot showing the down-regulation of NRP2 by shRNA (Fig. 2G). The immunoblot indicates a significant, but not complete down-regulation of NRP2 that is consistent with the significant decrease in tumour formation. We also note that the shRNA data were substantiated using a function-blocking NRP2 antibody (e.g. Fig. 2H).

2. "*What is the role of endogenous NRP2 in mice? Does the Ab block this as well as the human form? Why are mice not affected in Fig. 2H?*"

The NRP2 Ab (anti-Nrp2^{B}) used binds both human and mouse NRP2. It is a well-characterized antibody developed by Genetech that is currently in clinical trials. The effects of anti-Nrp2^{B} treatment on the normal adult mice have been reported previously (Caunt et al, 2008, *Cancer Cell*). Analysis of intestinal, cutaneous, pancreatic, and lymph node tissues by IHC of mice treated for five weeks with anti-Nrp2^{B} revealed no qualitative or quantitative differences between treated and untreated mice (Figure S6 of Caunt et al, 2008, *Cancer Cell*). These results are consistent with data indicating that NRP2 expression is negligible in most epithelia and the fact that we observed significant up-regulation of NRP2 in tumours (both human specimens shown in Figure 9 and TBP mouse model shown in Fig. 2).

3. "*Where is the proof of the specificity of the NRP-2 Ab? Can the authors provide rescue experiments in vivo without enhancing high GLI1 levels, for example by kd of SUFU as they suggest in the discussion*?"

As mentioned above, the anti-Nrp2^{B} antibody has been well-characterized by Genentech and currently being used in clinical trials. Importantly, it does not recognize NRP1. We also note in response to this comment that we used other approaches to implicate NRP2 including shRNA (e.g., Fig. 2G for tumour initiation and Fig. 3G for mammosphere formation) and an inhibitory peptide (Fig. 1G). The data obtained substantiate the specificity of the anti-Nrp2^{B} antibody.

We also performed experiments using SUFU shRNAs as requested. Our results suggest that Ras-induced GLI1 is predominantly dependent upon the ERK pathway as described by Stecca et al. (*PNAS*, 2007) and not on SUFU. We have included these new data as Figs. 7A, 7B and 7C.

4. "*Fig. 5B suggests inhibition of GLI1 protein function in a reporter assay by kd of NRP2. How is this thought to happen?*"

We propose that this is happening by inhibition of a FAK/Ras/ERK pathway as we discuss in the revised text.

5. "*The analyses with shSMO are interesting but a critical control is missing. Does shSMO inhibit GLI1? If so, it should inhibit the autocrine loop and block growth. Why does it not have this effect? Does shSMO inhibit mammosphere formation?*"

We performed the requested experiment and observed that down-regulation of SMO did not affect GLI1 expression (Fig. 6F) suggesting a predominant role of Ras in these triple negative breast cancer cells. As a positive control for shSMO, we show that SMO down-regulation in LNCaP prostate cancer cells inhibits GLI expression (Fig. 6C).

6. "*Conclusions with gain-of-function of GLI1 only can be tricky as this is a potent oncogene. The fact that FAK increases GLI1 expression does not mean that GLI1 mediates FAK function. More careful writing is needed*."

To clarify this point, the data we present demonstrate that CA-FAK increases GLI1 expression in SUM1315 NRP2^{low} cells (Fig. 5D) and that down-regulation of GLI1 decreases FAK activation in SUM1315 NRP2^{high} cells (Fig. 8G). These findings are consistent with our hypothesis that FAK/Ras signalling regulates GLI1 and the fact that GLI1 can induce NRP2 expression, which we have shown is able to promote α6β1-mediated FAK activation.

7. "*The section of RAS is misguided and misreferenced. Lauth and Toftgard is a review that does not directly address RAS work. Primary previous work has shown the GLI regulalton by RAS, AKT and MEK (for instance Steca et al., 2007; Varnat et al., 2009). Guan et al is not related to this topic. These papers have shown GLI1 is downstream of RAS in human cancer cells of various types*."

We have cited these references in the revised text and apologize for our oversight in not including these important papers.

8. "*It is not clear what is the level of GLI1 expressed in SUM1315 NRP2low cells. Too high levels can be toxic or induce a dominant oncogenic response that may not be related to epistatic effects. Again, performing such experiments with shSUFU or blockade of other endogenous factors that activate GLi1 may be more informative*".

The level of GLI1 expression in SUM1315 NRP2low cells is now shown in Figure 8B and it is comparable to endogenous GLI1 levels of SUM1315 NRP2 high cells (Fig. 8C). As mentioned above, Ras-induced GLI1 is predominantly dependent upon the ERK pathway and not on SUFU.

9. "*Why does overexpression of GLI1 lead to reduced adhesion to laminin but not collagen (Fig 7E-F_?)" Please note that overexpression of GLI1 increases adhesion to laminin (Fig. 8D)*".

We reported previously that NRP2 activates the α6β1 integrin, a specific laminin receptor, and that NRP2 does not regulate adhesion to collagen (Goel, *JCS*, 2012). Our finding that GL1

overexpression, which induces NRP2, increases adhesion to laminin but not collagen is consistent with these findings. Moreover, this is the first report demonstration the regulation of laminin adhesion by NRP2 in a GLI1-dependent manner.

10. "*It is important to resolve whether Gli1 is required for SUM1315 tumour growth in vivo. Is kd of GLI1 synergistic with the effects of the anti-NRP2 Ab*?"

We have included new data (Fig. 7H) showing the effect of GLI1 down-regulation, as well as NRP2 inhibition, on SUM1315 tumour growth. We observed comparable effects on tumour formation with either GLI1 down-regulation or NRP2 inhibition.

11. "*If NRP2 is causal for mammosphere formation and mammosphere cells cause tumour initiation, why does blockade of NRP2 only delay tumour growth in vivo (p.14)?*"

A total of 4 Ab injections were given to pregnant females and the mice were subsequently monitored for tumour occurrence. Based on the antibody half-life, a delay in tumour onset and not complete ablation is expected because transgenes (BRCA, T121 and p53) are continuously induced by WAP-Cre. Practically, it was not possible to maintain Ab treatment for such a long time. In the mammosphere assays, however, we were able we maintain the presence of the NRP2 Ab for the entire experiment.

12. "*What is the proof that GLI1 is RAS-dependent in vivo?*"

We provide new data that expression of CA-Ras increases tumour initiation (Fig 6G). Also, we now demonstrate that GLI1 down-regulation significantly inhibited mammosphere formation induced by CA-Ras (Fig. 6H).

13. "*The localization of GLI1 protein by Xu et al is largely unreliable*."

In response to this concern, we performed our own analysis of GLI1 expression using more than 30 triple-negative and 30 non-triple-negative breast tumours. Importantly, we used qPCR to obtain quantitative data. The results obtained reveal that triple-negative breast tumours express significantly more GLI1 than non-triple-negative breast tumours (Fig. 9D).

13. "*Additional references are required for the point of the role of Gli1 in cancer initiating cells (e.g. Clement et al., 2007; Peacock et al., 2007) and that Shh increases clonogenicity*."

We have cited these references in the revised text and apologize for our oversight in not including these key papers.

14- The mechanistic speculation needs a lot of work (p.15,16) incorporating previous works on GLI, ras, sufuh in multiple systems (e.g. Stecca et al., 2007; Varnat et al, 2009) and p53 (Abe et al., 2008; Stecca and Ruiz i Altaba, 2009). Care should also be taken equating mouse and human cell behaviour.

We have modified the Discussion to incorporate these suggestions.

© EMBO

15. "*It is also necessary to use the same cells in all assays. At this point it is not clear why are so many different cells used in the different assays*."

We believe that our multi-faceted approach of using cell lines, primary cell cultures, mouse models and human tumour specimens to draw conclusions is a strength of the study, as noted by the other reviewers. Nonetheless, we emphasize that all of the key findings were observed using SUM1315 cells.

Referee #2

"*For instance, the therapeutic testing of the blockade of the pathways should be done in a relevant breast cancer model, the xenograft model lacks some biological features of triple negative tumours that may impact in the treatment's outcome. The authors should perform a set of experiments in the TBP model and define the targeting specificity by looking the activation of downstream molecules of NRP2 and expression of GLI1 and Bmi1*."

We appreciate this comment but we note that we did use a mouse model of triple-negative breast cancer to test our hypothesis in the original submission. Specifically, we assessed the ability of a NRP2 antibody to impede tumour initiation in the TBP model (Fig. 2H) and we monitored the activation of GLI1 and BMI1 in TBP mice in response to NRP2 Ab treatment (Fig. 5I).

"*The mechanism controlling GLI1 should be further expanded, especially when the cell 2model used in the study are Hedgehog responsive*."

In response to this comment, we established that Ras-induced GLI1 expression is predominantly dependent upon the ERK pathway (Fig. 7A-C).

"*Is GLI2 involved in this regulatory mechanism*?"

Our data indicate that GLI2 is not involved in this regulatory mechanism because its expression is negligible in SUM1315 and five primary tumours as shown in the bar graph below. Moreover, there is no significant difference in GLI2 expression between NRP2^{high} and NRP2^{low} SUM1315 cells, in contrast to GLI1.

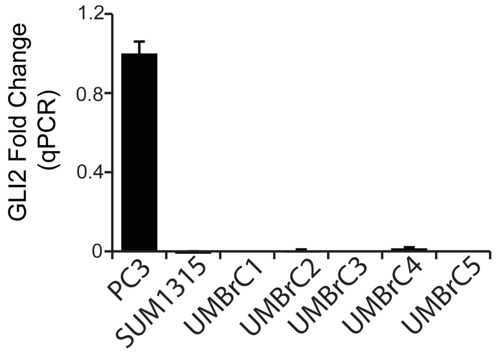

"*What are the factors binding to the GLI1 promoter to control its expression downstream of the NRP2 receptor?*"

This is an interesting question but as the editor noted in his decision letter, it is beyond the scope of this study.

"*In addition, the role of Ras in this process should be further validated, the dominant negative experiment is not ideal to define the role of this GTPase in the regulation of GLI1.*"

In response to this comment, we have added new data using Ras-shRNAs and a constitutively active Ras, as well as a constitutively active MEK. These new data are provided in Figs. 6F, 6G, 6H, 7A-C and 7H.

"*Finally, the expression of this newly identified pathway in triple negative breast cancer should be examined in clinical specimens. Are the components of the NRP2-VEGF-FAK-GLI1 expressed in triple negative tumours?*"

In response to this concern, we analysed NRP2, VEGF and GLI1 expression using more than 30 triple-negative and 30 non-triple-negative breast tumours. Importantly, we used qPCR to obtain quantitative data. The results obtained reveal that triple-negative breast tumours express significantly more of these molecules than non-triple-negative breast tumours (Fig. 9D). Also, we analysed the expression of phospho-FAK by immunoblotting extracts from 21 triple-negative and 21 non-triple-negative breast tumours. The densitometric analysis of these immunoblots is provided in Fig. 9E and it demonstrates that triple-negative breast tumours express significantly more phospho-FAK than non-triple-negative breast tumours. We also provide the original immunoblots here for the reviewer to analyse.

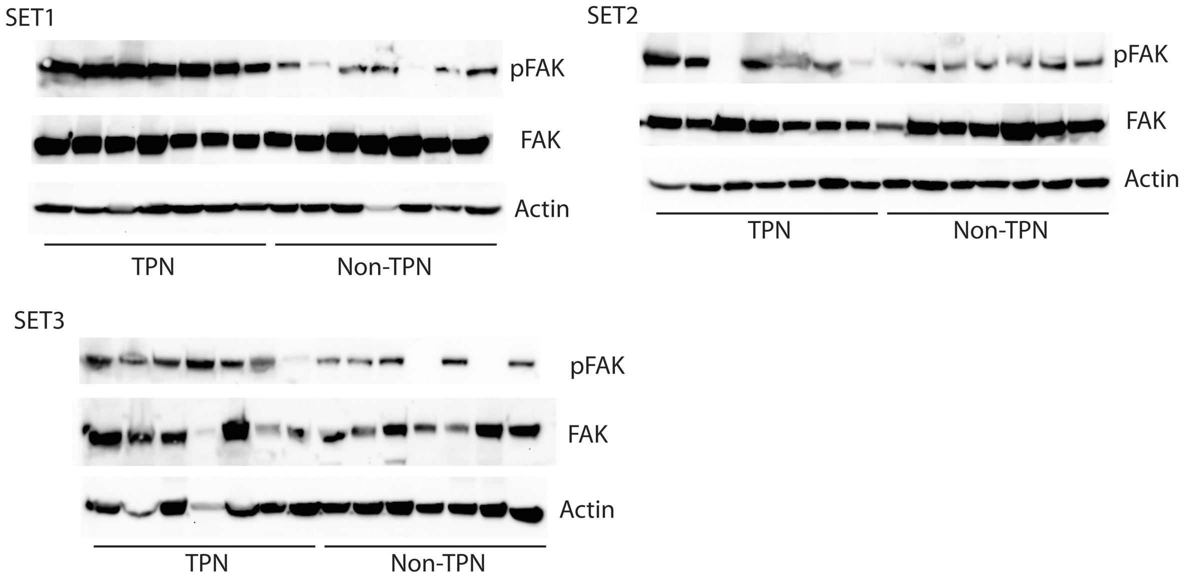

Referee #3 (General Remarks):

"*My concern regarding this manuscript is that there is a very strong slant towards cancer stem cells and promoting this autocrine mechanism as a cancer stem cell-specific mechanism. The manuscript is also plagued by some blanket statements regarding cancer stem cells that are simply not accurate. Most of the work describing breast cancer stem cells have relied on cell lines and the use of mammosphere assays - hardly a robust model system. A recent publication has challenged the use of sphere assays as a good indicator of in vivo tumourigenicity (see: Kim et al (2012) PNAS 109:6124-9). Likewise, in vivo transplantation assays are problematic since it has been demonstrated that normal luminal stem cells do not engraft efficiently in the absence of appropriate basal helper cells (Van Keymeulen et al (2011) Nature) - this latter observation could have major*

implications for the detection of cancer stem cells that have a luminal phenotype. My advise to the authors is to tone down the cancer stem cell aspect and steer clear of this controversy, because otherwise it will mar an otherwise pretty good manuscript."

In response to this concern, we have toned down the cancer stem cell aspect and the description of autocrine signalling as a cancer stem cell-specific mechanism.

Comments

Page 3: "*A distinguishing feature of TICs is their self-sufficiency and their use of autocrine and paracrine signalling pathways to sustain their function (DiMeo et al, 2009; Fillmore et al, 2010; Ginestier et al, 2010; Kim et al, 2012; Korkaya et al, 2011; Marotta et al, 2011; Sansone et al, 2007; Scheel et al, 2011)." This is a rather strange comment since I would expect that many different cell types in the body rely on autocrine/paracrine signalling for their functions and I hardly think that this is a property that distinguishes stem cells*".

We appreciate this comment and we have modified the text by deleting this statement.

"*When sorting out the NRP2^{high}/α6^{high} population from primary human tissue, are these NRP2^{high}/α6^{high} cells also gated on an epithelial marker such as EpCAM? My worry is that if it is just alpha 6 alone, could you not also be enriching for endothelial cells? Endothelial cells also express alpha 6 integrin, and presumably would have high levels of NRP2 expression*."

Yes, we used the EpCAM^{+} population for sorting for α6^{high} and /α6^{low} cells as described in the Methods section.

Page 6: "*The NRP2^{high}/α6^{high} population, which expressed high NRP2 (Fig. 1D), formed mammospheres significantly more than the NRP^{low}/α6^{low} population (Supporting Information Fig S1A)." Are the data in Fig S1A significantly different? If so, an asterisk should indicate this, or the wording changed*."

Yes, the data in Fig. S1A are statistically significant and we added an asterisk as requested.

"*Figure 1D dot plots: What is the background (fluorescence minus one or isotype control Ab) level of fluorescence these dot plots? This should be indicated*."

We added FACS profiles with an isotype control Ab in Fig. S1A.

Figures 5E: "*This is a very dramatic difference in the expression of Gli, Bmi1 and NRP2 between adherent cultures and sphere cultures (10-40X). How is this related to phenotype? Are adherent cells more luminal like? Are the sphere cells more basal/EMT like*?"

We have added new data that supports the reviewers' contention. Specifically, sphere cultures exhibit significantly decreased E-cadherin expression and increased expression of vimentin and fibronectin compared to adherent cultures (Fig. 5F).

Page 3, first paragraph: "*The hypothesis that breast tumours harbour a population of cells that has stem-like properties and can initiate tumorigenesis is supported by strong experimental evidence*

(Baccelli & Trumpp, 2012; Gupta et al, 2009; Keller et al, 2012; Korkaya et al, 2011)." I do not agree that Keller et al is an appropriate reference for the presence of breast tumour stem cells - this manuscript was about the cellular origins of different types of breast cancer. Al-Hajj et al would be a better reference."

We have included the Al-Hajj et al. reference as requested.

Page 3, first paragraph: "*The frequency of such tumour initiating cells (TICs) is high in poorly differentiated tumours (Pece et al, 2010) and these cells may be intimately associated with an epithelial mesenchymal transition (EMT) and contribute to metastasis (DiMeo et al, 2009; Mani et al, 2008; Scheel et al, 2011)." The statement regarding the relationship between stemness and EMT (basal cell phenotype) is not always the case. See: Kim J et al. Proc Natl Acad Sci U S A. 2012 Apr 17;109(16):6124-9*."

We have modified the text accordingly and included the Kim et al. study.

Figure 1A: "*Please show a flow dot plot showing how these luminal and stem cell populations were gated and sorted. Also, I would recommend that you call cell population not "luminal" or "stem", but instead label them according to their phenotype (e.g., CD44+/CD24-). It may be that the luminal cells have stem cell activity (see: Kim et al (2012) PNAS 109:6124-9), but you have not used the correct assay to detect this*."

The gating strategy for isolating luminal population ($CD44^+/CD24^+/EpCam^+$) and stem-like $CD44^+/CD24^-/EpCAM^+$ population was described previously by Gupta et al (*Cell*, 2011) and we have included the reference to this strategy in the Methods section. We also revised the labelling of Fig 1A as requested.

"*Graph in S1D: Is there not a significant difference between these two conditions? If so, an asterisk should indicate this*."

Yes, there is a significant difference and we have added an asterisk.

Page 7: "*We also established two cell lines from the ascites fluid of breast cancer patients. Both of these cell lines are able to form tumours in vivo (3 out of 5 mice after 135 days)." A sentence describing these tumours would be helpful (e.g., ER? HER2?). I would suggest deleting the 3 out of 5 mice and just state that these cell lines were tumourigenic in vivo. The frequency of tumour formation will be dependent on the cell dose and the conditions of the transplant (e.g., recipient mouse strain, inclusion of Matrigel in the transplant inoculumn.*"

We deleted the phrase "3 out of 5 mice" as requested and we have provided the ER/PR status for the ascites cells in the Methods section.

Page 7: "*These tumours, in marked contrast to MMTV-PyV-MT tumours, express abundant NRP2, which is localized on the surface of tumour cells (Fig. 2E)." How can you tell this from the image presented in Figure 2E? You would have to establish cell surface expression by flow cytometry, not by IHC*."

We have revised this statement by deleting the mention of surface expression.

© EMBO

Page 9: "*These findings are of particular interest since α6β1 (CD49f) is an established marker of many tumour stem-like cells including those of the breast (Lathia et al, 2010), and high expression of this integrin is a characteristic of TICs (Friedrichs et al, 1995; Honeth et al, 2008; Lathia et al, 2010; Mulholland et al, 2009; Schober & Fuchs, 2011; Vieira et al, 2012)." Need to be careful about making blanket statements. Many cells in the mammary gland express C49f (actually, most cells except the mature luminal cells in the normal breast express this protein), but not all of these cells are necessarily stem cells. Although stem cells express this marker, it is not a stem cell-specific marker*."

We have modified this text by stating that high α6 expression characterizes tumour initiating cells.

"*Materials and Methods: qRT-PCR methods appear to be missing*."

We have included the qRT-PCR method in the Materials and Methods section.

Figure 6A: "*This is relative fold-change...but to what?*"

Fold change was calculated relative to the value for Smoothened expression in SUM1315 cells, which was set at 1. We have included this description in the legend to Fig. 6A.

2nd Editorial Decision 15 January 2013

Thank you for the submission of your revised manuscript to EMBO Molecular Medicine. We have now received the enclosed reports from the referees whom we asked to re-assess it. You will be glad to see that the reviewers are now globally supportive and we can proceed with official acceptance of your manuscript pending the following minor changes:

- The description of all reported data that includes statistical testing must state the name of the statistical test used to generate error bars and P values, the number (n) of independent experiments underlying each data point (not replicate measures of one sample), and the actual P value for each test (not merely 'significant' or 'P < 0.05'). Please make sure that this if fully complied with.

- In addition, we noted some minor concerns regarding the figures: Please ensure that the resolution of the line graphs and western blots is improved and that labels are readable also at lower magnification. Also, it is important that PDF files are text searchable.

Please submit your revised manuscript as soon as possible, ideally within a week. I look forward to seeing a revised form of your manuscript as soon as possible.

***** Reviewer's comments *****

Referee #2 (General Remarks):

The revised version of the manuscript has significantly improved since the initial submission. The authors has been responsive to the reviewers' critiques, however, it is a pity that they do not further explore the regulatory mechanism controlling Gli1 activity. Especially, when this transcription factor is a central effector of NRP2 signaling during breast carcinogenesis.

Referee #3 (General Remarks):

The authors have addressed all of my concerns.

2nd Revision - authors' response 15 January 2013

We are submitting a revised version of our manuscript entitled “Gli1 Mediates Autocrine Signaling Involving Neuropilin-2 and the α6β1 Integrin that Contributes to Breast Cancer Initiation”.

We have responded to the editorial issues raised by the editors. Please note:

1. We provide the exact p-values, as well as the statistical tests used.
2. We increased the font size of the labels in the figures.
3. We provide the high resolution TIFF files.

We confirmed that the PDF files are text searchable
