# Supplementary material for: GLI1 regulates a novel neuropilin-2/α6β1 integrin based autocrine pathway that contributes to breast cancer initiation
Source: EMBO Mol Med. 2013 Feb 21;5(4):488–508. doi: 10.1002/emmm.201202078 (PMC3628099; doi:10.1002/emmm.201202078)
Supplement: Supplementary file 2 [file emmm0005-0488-sd2.pdf]

Fig. S1

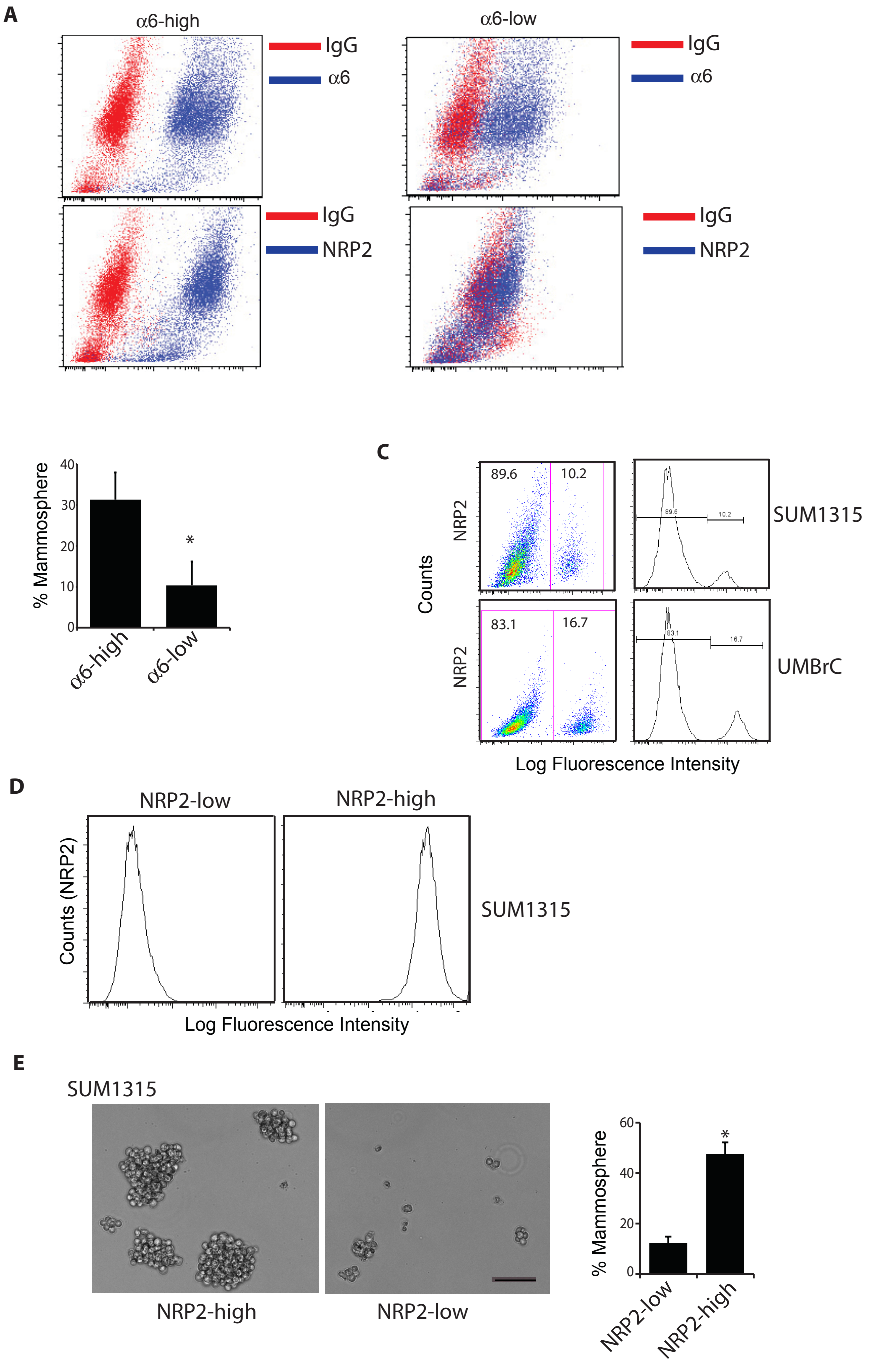
A
α6-high
α6-low
IgG
α6
IgG
NRP2
B
% Mammosphere
40
30
20
10
0
*
α6-high
α6-low
C
89.6
10.2
83.1
16.7
NRP2
Counts
SUM1315
UMBrC
Log Fluorescence Intensity
D
NRP2-low
NRP2-high
Counts (NRP2)
SUM1315
Log Fluorescence Intensity
E
SUM1315
NRP2-high
NRP2-low
% Mammosphere
60
40
20
0
*
NRP2-low
NRP2-high

Fig. S1. (**A**) UMBrC cells sorted into either $\alpha6^{high}$ or $\alpha6^{low}$ populations were analyzed for expression of NRP2 and $\alpha6$ using FACS analysis. (**B**) UMBrC cells sorted into either $\alpha6^{high}$ or $\alpha6^{low}$ populations were analyzed for their ability to form mammospheres. (**C**) $NRP2^{high}$ and $NRP2^{low}$ populations were sorted from SUM1315 and UMBrC cells by FACS using a NRP2 Ab. (**D**) SUM1315 cells sorted into $NRP2^{high}$ and $NRP2^{low}$ populations were re-analyzed for NRP2 surface expression using flow cytometry. (**E**) SUM1315 cells sorted into $NRP2^{high}$ and $NRP2^{low}$ populations were analyzed for their ability to form mammospheres (*p=0.01). Scale bar = 100 μm.
